# BitterDB database analysis plus cell stiffness screening identify flufenamic acid as the most potent TAS2R14-based relaxant of airway smooth muscle cells for therapeutic bronchodilation

**DOI:** 10.7150/thno.92492

**Published:** 2024-02-17

**Authors:** Kai Ni, Bo Che, Rong Gu, Chunhong Wang, Hongyang Xu, Huiduo Li, Shiyan Cen, Mingzhi Luo, Linhong Deng

**Affiliations:** Changzhou Key Laboratory of Respiratory Medical Engineering, Institute of Biomedical Engineering and Health Sciences, and School of Medical and Health Engineering, Changzhou University, Changzhou, Jiangsu, China.

**Keywords:** cell stiffness, bitter taste receptors, flufenamic acid, relaxation, anti-inflammation

## Abstract

**Rationale:** Bitter taste receptors (TAS2Rs) are abundantly expressed in airway smooth muscle cells (ASMCs), which have been recognized as promising targets for bitter agonists to initiate relaxation and thereby prevent excessive airway constriction as the main characteristic of asthma. However, due to the current lack of tested safe and potent agonists functioning at low effective concentrations, there has been no clinically approved TAS2R-based drug for bronchodilation in asthma therapy. This study thus aimed at exploring TAS2R agonists with bronchodilator potential by BitterDB database analysis and cell stiffness screening.

**Methods:** Bitter compounds in the BitterDB database were retrieved and analyzed for their working subtype of TAS2R and effective concentration. Compounds activating TAS2R5, 10, and 14 at < 100 μM effective concentration were identified and subsequently screened by cell stiffness assay using optical magnetic twisting cytometry (OMTC) to identify the most potent to relax ASMCs. Then the compound identified was further characterized for efficacy on various aspects related to relaxation of ASMCs, incl. but not limited to traction force by Fourier transform traction force microscopy (FTTFM), [Ca^2+^]_i_ signaling by Fluo-4/AM intensity, cell migration by scratch wound healing, mRNA expression by qPCR, and protein expressing by ELISA. The compound identified was also compared to conventional β-agonist (isoproterenol and salbutamol) for efficacy in reducing cell stiffness of cultured ASMCs and airway resistance of ovalbumin-treated mice.

**Results:** BitterDB analysis found 18 compounds activating TAS2R5, 10, and 14 at < 100 μM effective concentration. Cell stiffness screening of these compounds eventually identified flufenamic acid (FFA) as the most potent compound to rapidly reduce cell stiffness at 1 μM. The efficacy of FFA to relax ASMCs *in vitro* and abrogate airway resistance *in vivo* was equivalent to that of conventional β-agonists. The FFA-induced effect on ASMCs was mediated by TAS2R14 activation, endoplasmic reticulum Ca^2+^ release, and large-conductance Ca^2+^-activated K^+^ (BK_Ca_) channel opening. FFA also attenuated lipopolysaccharide-induced inflammatory response in cultured ASMCs.

**Conclusions:** FFA as a potent TAS2R14 agonist to relax ASMCs while suppressing cytokine release might be a favorite drug agent for further development of TAS2R-based novel dual functional medication for bronchodilation and anti-inflammation in asthma therapy.

## Introduction

Asthma is an obstructive airway disease affecting more than 300 million people worldwide, which is characterized by airway inflammation, airway remodeling, and airway hyperresponsiveness (AHR) 1-3. AHR is instigated by excessive contraction of airway smooth muscle cells (ASMCs), which narrows the airway lumen and thereby leads to obstruction of airflow 4, 5. Given the key roles of ASMCs in AHR, bronchodilators are usually prescribed as the first-line medications for treating asthma 6.

Currently, β_2_-adrenergic receptor (β_2_AR) agonists, also called “β-agonists” are the conventional U.S. Food and Drug Administration (FDA)-approved bronchodilators. However, these currently available β-agonists have been reported for failure of adequate control of asthma symptoms for more than half of asthmatic patients according to several clinical trials 7-9. It has also been shown that β-agonists can cause deleterious side effects, which increase bronchial hyperreactivity and the risk of morbidity 10, 11. In addition, β-agonists are often combined with corticosteroids in the clinical treatment of asthma to suppress airway inflammation, but corticosteroids are also known to increase the risk of respiratory infections and thus cause mild acute symptoms such as dysphonia and thrush or severe symptoms 12. Hence, there is an unmet clinical need to find alternative pharmaceutic agents that can relax ASMCs for bronchodilation, and preferably also control inflammation.

Recently, G protein-coupled bitter taste receptors (TAS2Rs) are highly expressed in ASMCs. And unlike in the tongue for protection against toxins, TAS2Rs in ASMCs mediate relaxation of the cells, indicating a great potential as novel therapeutic targets of bronchodilation 13-16. Furthermore, it has been elucidated that TAS2R activation initiates Ca^2+^ signaling with a cascade reaction of G protein, phospholipase C β2 (PLCβ2), and inositol trisphosphate (IP_3_), which leads to marked relaxation of ASMCs and bronchodilation 8, 17.

However, no TAS2R-based drug has been approved yet as a bronchodilator for asthma treatment, because most TAS2R agonists screened so far display relatively low potency for activation of the TAS2R signaling pathway 18, 19. Such low potency in the mid-to-high micromolar range makes sense in the case of taste perception in the tongue, but it is disadvantageous for relaxing ASMCs in the lung and thus limits clinical approval 8. Nevertheless, some databases contain thousands of chemical compounds identified as bitter substances with specific chemical features associated with bitterness 20-23. The largest among them is BitterDB that holds 1041 bitter taste compounds and each chemical compound can be searched online to predict its bitterness by chemoinformatics analysis 20. With such a large repository of bitter taste substances in BitterDB available for free evaluation, we believe that there is a strong possibility for us to find potent bitter agonists for relaxing ASMCs.

Therefore, in this study we systematically searched BitterDB for bitter compounds that met the requirement of relaxing ASMCs at low effective concentrations (< 100 μM). Subsequently, we evaluated these bitter agonists chosen from BitterDB for the efficacy of relaxing ASMCs in both cell culture and animal model, in terms of their ability to alter cell stiffness, contractility, and so on as assessed by techniques such as optical magnetic twisting cytometry (OMTC) and Fourier transform traction force microscopy (FTTFM). Eventually, in the entire repository of BitterDB we found that flufenamic acid (FFA) could exert rapid relaxation of cultured ASMCs at 1 μM, while being equivalent to β-agonists in reducing airway resistance in ovalbumin-induced asthmatic mice. Then, we verified that FFA mediated the relaxation of ASMCs by activating TAS2R14 signaling pathway and opening large-conductance Ca^2+^-activated K^+^ (BK_Ca)_ channels. Moreover, we found that in addition to causing relaxation, FFA could also attenuate lipopolysaccharide (LPS)-induced inflammatory response in cultured ASMCs. These findings together indicate that FFA as a TAS2R14 agonist might be the best candidate to be further developed as a novel TAS2R-based drug with dual-function of bronchodilation and anti-inflammation for asthma therapy.

## Materials and Methods

### Cell cultures, animals, and reagents

Primary cultured human ASMCs (#BNCC339826) were purchased from BeNa Culture Collection (Beijing, China) and used at passages 4-8. Cells were grown in the Dulbecco's modified Eagle's medium (DMEM) supplemented with 10% fetal bovine serum (FBS), 2 mM L-glutamine, 100 units/mL penicillin, and 100 μg/mL streptomycin in an incubator containing 5% CO_2_ humidified at 37 °C. The cells showed a typical “hill and valley” appearance under phase-contrast microscopy and physiological response to agonists.

Female BALB/c mice (6-8 weeks of age; 20-22 g) that had been maintained in a specific pathogen-free environment were purchased from Cavens Lab Animals Co. Ltd (Changzhou, China), which is an authorized supplier of experimental animals for medical research [license No. SCXK-(JS)-2021-0013]. All animal experiments were approved by the Animal Care and Use Committee of Changzhou University in China (Proof No. 2022022804).

Drugs including kaempferol (#K107144), quinine (#Q105030), naringenin, (#N107346), benzoin (#B100909), tangeretin (#T123655), apigenin (#A106675), quercetin (#Q111273), chloramphenicol (#C100334), chlorhexidine (#C101647), clonixin (#C153377), mefenamic acid (#M157895), genistein (#G106673), niflumic acid (#N129597), pentagalloylglucose (#P115686), 1,10-phenanthroline (#P425360), cucurbitacin E (#C305256), and denatonium benzoate (#D124654) were obtained from Shanghai Aladdin Bio-Chem Technology Co., Ltd. (Shanghai, China). FFA (#N865659) was purchased from Macklin (Shanghai, China). Reagents including U73122 (#HY-13419), 2-APB (#HY-W009724), BAPTA-AM (#HY-100545), thapsigargin (#HY-13433), paxilline (#HY-N6778) and dexamethasone (Dex, #HY-14648) were purchased from MedChemExpress (MCE, Shanghai, China). 6-methylflavanone (6-Met, #M1403) was obtained from TCI Development Co., Ltd. (Shanghai, China). Transferrin (#T8158) and insulin (#91077C) were purchased from Sigma-Aldrich (St. Louis, MO, USA). LPS (#S1732) was obtained from Beyotime Biotechnology (Shanghai China). Collagen type I (#5279) was purchased from Advanced BioMatrix (Poway, CA, USA). DMEM (#11885092), FBS (#16000-044), penicillin-streptomycin (#15140122), and trypsin (#25200056) were purchased from Thermo Fisher Scientific (Waltham, MA, USA). Cell culture flasks and plates were purchased from Corning Inc. (Corning, NY, USA).

### Identification of bitter compounds from BitterDB for relaxation of ASMCs

BitterDB (http://bitterdb.agri.huji.ac.il) is a database that gathers information about bitter-tasting natural and synthetic compounds, and their cognate bitter taste receptors (T2Rs or TAS2Rs). It currently holds 1041 bitter compounds obtained from over 100 scientific publications and their associated bitter receptors from Human, Mouse, Chicken, and Cat, and is the world's largest publicly available bitter compounds collection for research. BitterDB provides several ways to search for bitter compounds by different criteria and for bitter molecules with structures similar to a query compound. Therefore, in this study, we first searched BitterDB for bitter compounds that could be used as drug agents for bronchodilation. Considering that ASMCs are known to mainly express the TAS2R5, 10, and 14 subtypes of TAS2Rs, we first searched BitterDB for bitter compounds that activate TAS2R5, 10, and 14. Then we compared and sorted those resultant bitter compounds by their effective concentrations, leading to the identification of the bitter compounds that could activate TAS2R5, 10, and 14 with low effective concentrations (< 100 μM). Specifically, we chose bitter compounds that could effectively activate TAS2R5 and TAS2R10 at a concentration below 100 μM, and TAS2R14 at a concentration below 10 μM, for further evaluation of efficacy to relax ASMCs. If there were multiple compounds found in BitterDB with the same effective concentration, only one was chosen for the following experiments.

### Cell stiffness screening of the BitterDB-identified bitter compounds for the most potent one to relax ASMCs

The bitter compounds chosen from BitterDB following the above process were then screened for their potency to relax ASMCs in terms of cell stiffness reduction of cultured ASMCs. We measured the cell stiffness of cultured ASMCs by using OMTC as described previously 24-27. In brief, Arg-Gly-Asp-coated ferrimagnetic microbeads (4.5 μm diameter) were added to ASMCs plated for 12 h on type I collagen-coated rigid dishes (96-well plate, Immunon II) for 30 min. Subsequently, the cells were washed with a serum-free medium to remove unbound beads. Then the microbeads were magnetized horizontally with a brief 1,000-Gauss pulse and twisted in a vertically aligned homogenous magnetic field (20 Gauss) that was varying sinusoidally in time.

To evaluate the variation of cell stiffness in response to drug treatment, the cells were first measured for 60 s to achieve baseline stiffness. Then a drug (100 μM) was added to the cells, after which the cells were continuously measured for stiffness for up to 330 s. Measurements were performed at a single frequency of 0.75 Hz. The dynamic cell stiffness response of ASMCs to the drug was quantified by the ratio of the averaged plateau value of cell stiffness measured in the presence of the drug treatment (G_1_) to the baseline stiffness measured in the absence of the drug treatment (G_0_), which was expressed as the cell stiffness reduction rate, i.e. (G_0_ - G_1_)/G_0_. And the values of (G_0_ - G_1_)/G_0_ resulting from exposure to different bitter compounds were compared to determine the most potent bitter compound to relax ASMCs (here after referred to as the bitter compound) for further evaluation.

### Assessment of the bitter compound's ability to reduce traction force of ASMCs

Subsequent to cell stiffness screening, the bitter compound identified as most potent was further evaluated for its ability to reduce traction force of cultured ASMCs. We measured traction force of cultured ASMCs by using FTTFM as described previously 16, 28-30. Briefly, the cells were seeded onto polyacrylamide gel embedded with fluorescent microbeads (0.2 μm, #F8810, Molecular probes, Eugene, OR, USA) coated with type I collagen (0.1 mg/mL) in serum-free medium for 12 h (5,000 cells/dish). Prior to treatment with the bitter compound, the field of view for microscopic imaging was selected with a single cell; then control images of the cell and the cell-free microbeads position were recorded by phase-contrast and fluorescence microscopy, respectively. The cell-free (traction-free) microbead position was used as a reference point for bead displacement. Subsequently, the bitter compound was added to the cell culture and the bead positions were recorded at given time points. After the drug treatment, the cell was trypsinized and removed before the microbead position was imaged again. Thus, the displacement of the microbead from the reference point induced by the cell (i.e. the substrate deformation) was converted to the change of cell traction force on the substrate using proprietary Matlab software (MathWorks Corp., Natick, MA, USA) according to the images of the cell (phase contrast) and the microbeads (fluorescence) obtained before and after the drug treatment.

### Assessment of the bitter compound's effect on Young's modulus of ASMCs

In addition to traction force, we also evaluated the effect of the bitter compound on Young's modulus of ASMCs. Young's modulus of the cell was characterized at the nanometer scale by using a Nanowizard II atomic force microscope (AFM, JPK Instruments AG, Berlin, Germany) mounted on an Olympus IX 81 inverted light microscope as described previously 24. Briefly, after ASMCs were seeded on a Petri dish for 24 h, the medium was changed with a serum-free culture medium. Then the bitter compound was added to the cell culture for 5 min, the dish was mounted in a Petri dish heater (JPK Instruments) set at 37 °C. For detecting Young's modulus, the tip of the cantilever was pushed into the cell surface and the indentation depth and the deflection of the cantilever were recorded. Then, Young's modulus was obtained by fitting the force-indentation curve to the Hertz model, where force was determined by multiplying the deflection by the spring constant of the cantilever following Hooke's law 31.

### Assessment of the bitter compound's effect on migration of ASMCs

Effect of the bitter compound on migration of cultured ASMCs was also evaluated by using a modified scratch-wound healing method as previously described, which might reveal the additional role of the bitter compound in asthma treatment since migration is another important mechanical behavior of ASMCs primarily in association with pathogenic airway remodeling 32, 33. Briefly, a high density of cells (2×10^5^ cells/cm^2^) were inoculated into 6-well plates (Corning) and reached a confluent monolayer. Subsequently, an experimental wound was made using a sterile micropipette tip, then the cells were washed 3 times with sterile phosphate buffered saline (PBS) and then added with the bitter compound. Wound areas were observed and recorded at 0, 12, and 24 h by using a Cell Observer System (Zeiss, Germany) equipped with a CO_2_ and temperature control chamber. The experimental wound area was quantified manually using “Area measurement” in ImageJ software and normalized to the wound area at the start of the experiment, and the migration distance was defined by the ratio of the wound healing area to the length of the wound area. ImageJ software and Matlab (The MathWorks) were used to analyze the migration rates and trajectories of the ASMCs.

### Assessment of the bitter compound-induced intracellular calcium signaling ([Ca^2+^]_i_) in ASMCs

To evaluate intracellular response of ASMCs to the bitter compound stimulation, intracellular calcium signals were visualized using the membrane-permeable [Ca^2+^]_i_-sensitive fluorescent dye Fluo-4 acetoxymethylester (Fluo-4/AM, #F14201, Thermo, MA, USA), as described previously 34. Briefly, cultured ASMCs were inoculated (~10^4^ cells per dish) into a confocal Petri dish with a glass bottom. Next, the cells were incubated with 5 μM Fluo-4/AM for 30 min at 37 ˚C in a 5% CO_2_ incubator. The cells were then washed with Tyrode's solution and incubated for 20 min to allow complete de-esterification of the cytosolic dye. The fluorescence intensity of the sample was measured by laser scanning confocal microscopy (LSM710, Carl Zeiss, Jena, Germany) using an excitation wavelength of 488 nm and an emission wavelength of 505 nm, which represented the influx of the [Ca^2+^]_i_. Subsequent image processing and analysis were performed offline using ImageJ software. Baseline Fluo-4 fluorescence (F_0_) was determined by averaging the first 10 frames of each experiment. [Ca^2+^]_i_ was represented as F/F_0_, with F calculated by integrating Fluo-4 over entire cells for global [Ca^2+^]_i_.

### Assessment of bitter compound's effect on BK_Ca_ channels in ASMCs

To evaluate the effect of bitter compound on currents of BK_Ca_ channels involving in bitter compound-induced relaxation of ASMCs, BK_Ca_ outward currents of the cultured ASMCs were determined with whole-cell patch clamp recording, as described previously 35, 36. The bath solution contained the following: NaCl 145 mM, KCl 4 mM, CaCl_2_ 2 mM, MgCl_2_ 1 mM, Glucose 10 mM, and HEPES 10 mM (pH adjusted to 7.2 with KOH). The pipette solution consisted of KCl 140 mM, EGTA 0.05 mM, MgCl_2_ 1 mM, Na_2_ATP 4 mM, and HEPES 10 mM (pH adjusted to 7.4 with KOH). Potassium currents were performed using a Multiclamp 700B amplifier (Molecular Devices, San Jose, CA, USA). Cells were voltage clamped with 500 ms voltage steps from -50 to +70 mV in 10 mV increments from a holding potential of -70 mV. BK_Ca_ currents were verified by the use of paxilline, a BK_Ca_ inhibitor.

### Assessment of the bitter compound's effect on stress fiber distribution of ASMCs

Intracellular structural remodeling of ASMCs in response to the bitter compound was evaluated by fluorescent microscopy of F-actin cytoskeleton before and after drug treatment. For immunofluorescent staining, ASMCs were cultured in the absence or presence of the bitter compound for 24 h, followed by cell fixation with 3.7% paraformaldehyde for 15 min at room temperature (RT) and cell permeabilization with 0.5% Tween-X-100 for 15 min at RT. Nonspecific antibody binding was blocked by incubating the samples in PBS containing 1% bovine serum albumin (BSA) for 1 h at RT. F-actin was stained with fluorescent red phalloidin (#R415, Thermo Fisher, MA, USA) for 20 min and nuclei were stained with blue DAPI (#C1005, Beyotime, Shanghai, China) for 3 min in the dark. Between all staining steps, cells were washed 3 times with PBS. Stained cells were visualized by using laser scanning confocal microscopy at ×40 objective (LSM710, Zeiss, Germany). Acquisition parameters were kept constant during the experiments.

### Assessment of siRNA transfections

To knock down TAS2R14, ASMCs (70 to 80% confluent) in six-well plates were incubated with a mixture of TAS2R14 siRNA (50 nM), Lipofectamine 3000, and Opti-MEM (#31985070, Gibco, USA) for 48 hours. Control cells were treated with scrambled siRNA and transfection reagent. TMEM16A silencing was performed using the method described above. siRNA for TAS2R14 and TMEM16A were obtained from RiboBio, China.

### Assessment of the bitter compound's effect on mRNA expression

Effect of the bitter compound on mRNA expression of key genes associated with relaxation and inflammation cytokines of ASMCs was evaluated with real-time quantitative PCR. Total RNA from cultured ASMCs was extracted using the TRI Reagent RNA Isolation Reagent (#T9424, Sigma-Aldrich, St. Louis, MO, USA) and the extracted RNA was quantified on Nanodrop 2000 spectrophotometer (Thermo Scientific, Willmington, DE, USA). 500 ng total RNA was used to generate 1st strand cDNA using the Revert Aid First Strand cDNA Synthesis Kit (#K1622, Thermo, MA, USA). Primers for key genes associated with relaxation of ASMCs including contractile protein: TAS2R14 (Forward: 5'-CATACCCTTTACTTTGTCCCTG-3', Reverse: 5'-AGGATAAGCCATTCCCATCACC-3'), TMEM16A (Forward: 5'-GAGCCGCCGTGGTCGGAAAA-3', Reverse: 5'-GGGAGAGGGTGCCCATCGGT-3'), calponin (Forward: 5'-AGGTGAACGTGGGAGTG AAG-3', Reverse: 5'-GGCTGGCAAACTTGTTGGTG-3'), cytokines: interleukin-1β (IL-1β) (Forward: 5'-CTGAGCTCGCCAGTGAAATG-3', Reverse: 5'-TGTCCATGGCCACAACAACT-3'), IL-2 (Forward: 5'-TGCTGATGAGACAGCAACCA-3', Reverse: 5'-GGCCTGATACGTTTTAAGTGGG-3'), IL-6 (Forward: 5'-CCAGAGCTGTGCAGATGAGT-3', Reverse: 5'-AGTTGTCATGTCCTGCAGCC-3'), tumour necrosis factor-α (TNF-α) (Forward: 5'-TGAAAGCATGATCCGGGACG-3', Reverse: 5'-CAAAGTGCAGCAGGCAGAAG-3'), interferon gamma inducible protein-10 (IP-10) (Forward: 5'-AACTGTACGCTGTACCTGCAT-3', Reverse: 5'-GCATCGATTTTGCTCCCCTC-3'), and monocyte chemoattractant protein-1 (MCP-1) (Forward: 5'-CATTGTGGCCAAGGAGATCTG-3', Reverse: 5'-CTTCGGAGTTTGGGTTTGCT T-3'), and GADPH (Forward: 5'-TGACGCTGGGGCTGGCATTG-3', Reverse: 5'-GGCTGGTGGTCCAGGGGTCT-3') as the reference gene were purchased from General Biosystems (Anhui, China). PowerUp SYBR Green Master Mix (#A25742, Applied Biosystems, CA) was used. The reaction was run in the qRT-PCR system (StepOnePlus, Applied Biosystems) using 1 µL of the cDNA in a 10 µL reaction according to the manufacturer's instructions in triplicate. Calibration and normalization were done using the 2^-∆∆CT^ method, where ∆∆C_T_ = C_T_ (target gene) -C_T_ (reference gene). Fold changes in mRNA expression were calculated from the resulting C_T_ values from three independent experiments.

### Assessment of the bitter compound's effect on cAMP level of ASMCs

Effect of the bitter compound on intracellular cAMP level of ASMCs was evaluated with ELISA. ASMCs were treated with the bitter compound for up to 30 min. Subsequently, the cells were subjected to ultrasonic lysis in the presence of the phosphodiesterase inhibitor IBMX (10^-5^ M, #HY-12318, MCE, Shanghai, China) in PBS and centrifuged at 14000 rpm (18407 g) for 10 min under 4 °C. Then the level of cAMP was detected by ELISA using the cAMP ELISA kit (#ab65355, Abcam, USA) following the manufacturer's protocol. Absorbance at 450 nm was measured using a colorimetric 96-well plate reader (Infinite F50, Tecan, Zürich, Switzerland). Data were expressed as ratios of the optical density of groups treated with the bitter compound to that of vehicle control groups.

### Assessment of the bitter compound's effect on viability of ASMCs

Effect of the bitter compound on viability of cultured ASMCs was finally evaluated by using the cell counting Kit-8 (CCK-8) assay kit (#C0037, Beyotime Biotechnology, Shanghai, China) as described previously 37. Cells were plated in 96 well plates at a density of 1×10^4^ cells/well. After overnight cultivation, the bitter compound was added, and then 10 μL of CCK-8 solution was added to the cells for 4 h at 37 °C. Absorbance was acquired by using an automatic microplate reader at 450 nm (Infinite F50, Tecan, Männedorf, Switzerland).

### Assessment of the bitter compound's *in vivo* effect on lung histopathology, inflammation, and function in ovalbumin-induced mouse models of asthma

After an *in vitro* assessment of the bitter compound's effects on ASMCs, we tested the *in vivo* effect of the bitter compound on lung histopathology and inflammation in ovalbumin (OVA)-induced mouse models of asthma using BALB/c mice. BALB/c mice with ovalbumin-induced asthmatic lung pathologies (OVA group) were prepared as described previously 38, 39. Briefly, female BALB/c mice 6-8 weeks of age were intraperitoneal injections (I.P.) injected with 100 µg of OVA (#A5503, Sigma-Aldrich MO, USA) and 2 mg aluminum hydroxide in 200 µL PBS on day 1, 7 and 14, respectively. From day 15, the mice were placed in a closed plastic box (25 cm × 21 cm × 16 cm) and were challenged with an aerosolized 1% OVA solution in PBS for 30 min once daily for 7 days using an ultrasonic air nebulizer (INQUA NEB plus PARI GmbH, Starnberg, Germany; 350 mg/min). The control group of mice received 200 µL of 0.9% saline solution via I.P. injection and were challenged with aerosolized 0.9% saline solution on the same days as the OVA group.

Twenty-four hours after the last challenge, the mice were evaluated for lung histopathology, inflammation, and function (airway resistance). Lung tissue slices from the OVA-challenged mice were stained with hematoxylin and eosin (HE) to investigate the inflammatory histopathological changes in the lungs of the mice. Briefly, the mouse's right lung was excised and immersed in fresh 4% formalin for ≥ 24 h. Then the lung was cleared, dehydrated in a graded series of ethanol solutions, and infiltrated in the xylene solution. The tissues were embedded in paraffin and cut into 10-μm-thick sections. Each section was placed on a glass slide, stained with HE, and photographed under a light microscope (Nikon, Japan) at x40 objective for morphological analysis and examination of cell infiltration.

Bronchial alveolar lavage fluid (BALF) was also collected from the mouse and subjected to centrifugation. Cell pellets were suspended in 1 mL PBS to proceed with total and differential cell counts. The total number of cells was determined by hemocytometer and data was expressed as cells/mL. For the differential cell count, cells deposited on slides were stained using the Wright-Giemsa method and counted by a skilled observer blinded to experimental details. The percentages of eosinophils, neutrophils, lymphocytes, and macrophages were calculated by counting 200 cells for each slide at ×40 objective.

Airway resistance (Rrs) of the mouse was measured using a computer-controlled FlexiVent system (SciReq, Montreal, PQ, Canada) as described previously 38, 40. Briefly, the mouse was anesthetized and the trachea was intubated and connected to the adapter of the instrument. After calibration and 5 min adaptation, the mouse was nebulized with 30 µL saline containing methacholine (MCh, #HY-A0083, MCE, Shanghai, China) at incremental doses (0, 1.56, 3.125, 6.25, 12.5, 25, and 50 mg/mL) for 30 s followed by 3 min rest repeatedly, until the sustained airway resistance became ∼ 5 to 6 -fold greater than baseline. Then the mouse inhaled the bitter compound at different dosages, and 3 μg salbutamol (#HY-B1037, MCE, Shanghai, China) as a conventional short-acting β_2_-adrenergic agonist treatment. Results were expressed as the percentage of airway resistance after treatment with the respective drug (MCh, MCh + salbutamol, MCh + bitter compound) at each dose relative to the baseline value.

### Statistical analysis

Statistical analysis was performed by using GraphPad Prism (GraphPad Software, La Jolla, CA). Unless stated otherwise, data were reported as means ± SD, and n represented the number of mice for animal test or the number of independent experiments for cellular test, respectively. Comparisons between multiple groups were analyzed using one-way ANOVA with a Tukey post hoc test and two-group comparisons were analyzed using the Student's *t* test. A value of *p* < 0.05 was considered to indicate a statistically significant difference.

## Results

### BitterDB database search and analysis identified 18 bitter compounds that may relax ASMCs at a low effective concentration

BitterDB database contains 1041 bitter compounds and 25 different subtypes of bitter taste receptors, which are available for evaluation to meet specific requirements of intended therapeutic purpose. Considering that ASMCs highly express TAS2R5, 10, and 14 subtypes for critically mediating cell relaxation, we reasoned that among the 1041 bitter compounds in the BitterDB database, only those binding to TAS2R5, 10, and 14 subtype receptors may meet the requirement of relaxing ASMCs at low concentration 41. Therefore, we searched and analyzed the whole BitterDB database of 1041 compounds and 25 subtype receptors, and found 199 compounds that could activate TAS2R5, 10, and 14 receptors. Among the 199 compounds, those acting as TAS2R5, 10, and 14 agonists accounted for 3%, 22%, and 85%, respectively.

In the BitterDB database, the lowest concentration for a compound to generate a detectable calcium signal was designated as the compound's effective concentration and used as a measure of potency for the compound as a bitter agonist because calcium increase is known to be required for a bitter compound to induce relaxation of ASMCs 18. Therefore, we further narrowed down the range of the bitter compounds that could be used for bronchodilation, by comparing and sorting the 199 bitter compounds according to their effective concentrations (**Table S1**). These effective concentrations ranged from 0.01 to 30000 μM, indicating a possibility of finding suitable compounds to meet the requirement of relaxing ASMCs at a low effective concentration.

**Figure 1:**
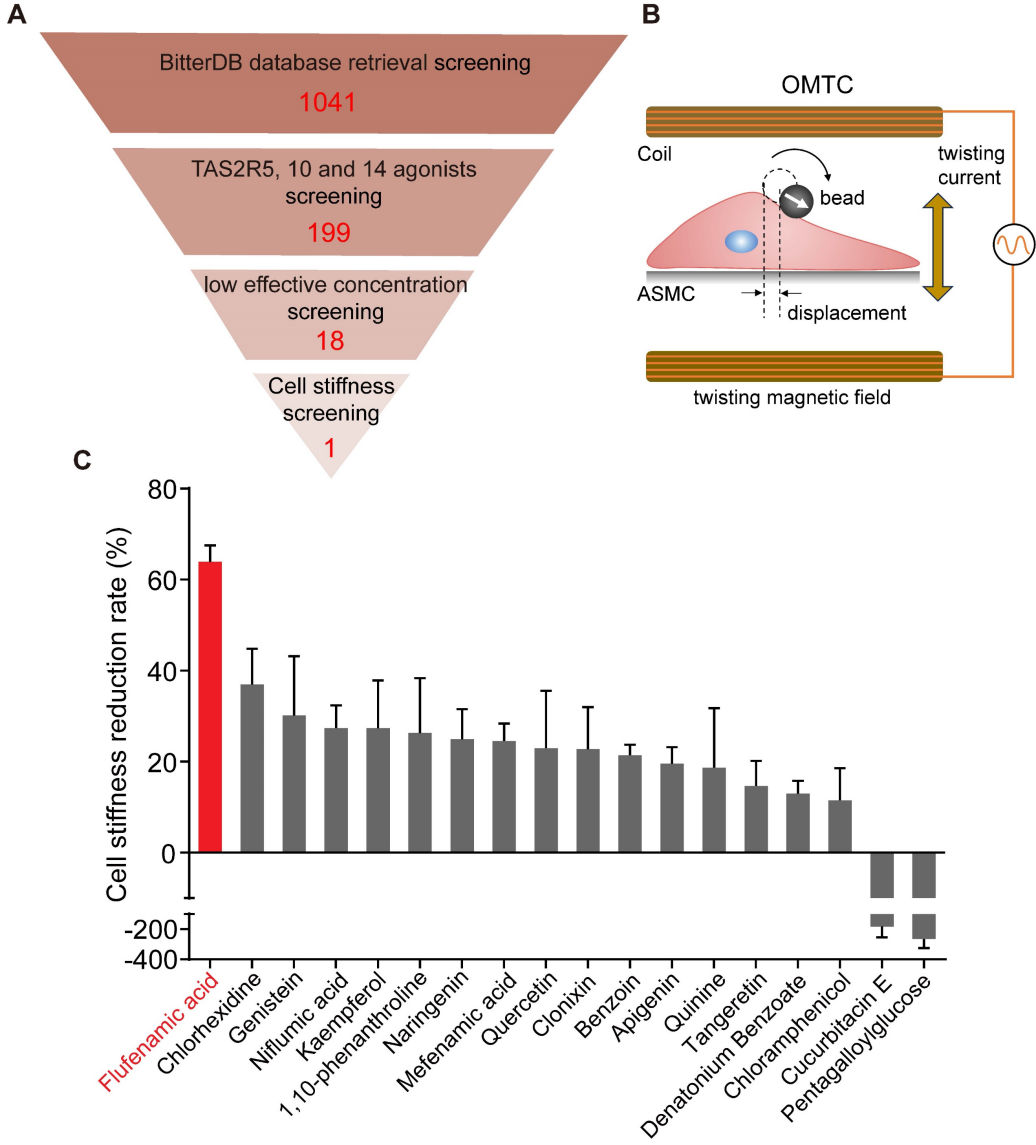
** Identification of the most potent bitter compound for relaxing ASMCs based on analysis of BitterDB database and cell stiffness screening.** (**A**) Schematic diagram of the processing steps of BitterDB database search and analysis plus cell stiffness screening to identify the most potent bitter compound for relaxing ASMCs. (**B**) Schematic diagram of cell stiffness measurement by OMTC. (**C**) The cell stiffness reduction rate of ASMCs induced by each of the 18 bitter compounds at 100 μM (n = 3).

Consequently, we selected the compounds with effective concentrations below 100 μM as suitable candidates to be functionally/mechanically evaluated for efficacy in relaxing ASMCs. Specifically, we found only pentagalloylglucose and 1,10-phenanthroline had effective concentrations below 100 μM as TAS2R5 agonists. For TAS2R10 and 14, multiple compounds had the same low effective concentration, and we chose only one of them for further evaluation. As a result, we chose cucurbitacin E, denatonium benzoate, quinine, benzoin, and chloramphenicol (effective concentration < 100 μM) as TAS2R10 agonists for further evaluation. Considering the largest proportion of compounds in **Table S1** were TAS2R14 agonists, we used effective concentration below 10 μM as the selection requirement for TAS2R14 agonists to further narrow down the selected range. Eventually, we chose flufenamic acid, chlorhexidine, tangeretin, kaempferol, quercetin, clonixin, mefenamic acid, genistein, niflumic acid, apigenin, and naringenin as TAS2R14 agonists for further evaluation. Thus, as shown in **Table 1** and** Figure 1A**, we identified altogether 18 bitter compounds that could activate TAS2Rs at low effective concentrations, which were further evaluated for their potency to relax ASMCs as described below.

### Cell stiffness screening identified flufenamic acid as the most potent bitter compound for relaxation of ASMCs

The 18 bitter compounds selected above were then tested at 100 μM for their ability to reduce cell stiffness of ASMCs by using OMTC, which led to the identification of the most potent bitter compound for relaxing ASMCs. During OMTC, ferrimagnetic bead tagged on ASMC was twisted to move in displacement due to the torque induced by a magnetic twisting field, and the ratio of torque to displacement measures cell stiffness, as shown in the diagram (**Figure 1B**). Furthermore, the cell stiffness of ASMCs measured by OMTC is known to decrease in response to relaxation drug treatment, which has been found to correlate with the relaxation of ASMCs measured by other techniques such as FTTFM 30. Since cell stiffness was technically easier and more efficient to measure a large number of cells, it is often used as a surrogate of potency test for drugs to relax ASMCs.

The results of cell stiffness screening and sorting of the 18 bitter compounds are shown in **Figure S1.** It could be seen that except for cucurbitacin E and pentagalloylglucose, most of the 18 bitter compounds at 100 μM could immediately decrease cell stiffness. The cell stiffness reduction rate of ASMCs after being treated respectively with the 18 bitter compounds was shown in **Figure 1C**. Clearly, in terms of their potency to relax ASMCs (i.e. the compound-induced cell stiffness reduction rate), the 18 compounds ranked in the order of flufenamic acid (FFA) > chlorhexidine > genistein > niflumic acid > kaempferol > 1,10-phenanthroline > naringenin > mefenamic acid > quercetin > clonixin > benzoin > apigenin > quinine > tangeretin > denatonium benzoate > chloramphenicol > cucurbitacin E > pentagalloylglucose. These data indicate that FFA displayed maximal efficacy or in other words was the most potent among the 18 selected bitter compounds to relax ASMCs, suggesting that it may function at a low micromolar dosage to relax ASMCs.

### Cell stiffness-characterized dose-effect relationship demonstrated that flufenamic acid could rapidly relax ASMCs at 1 µM

**Figure 2:**
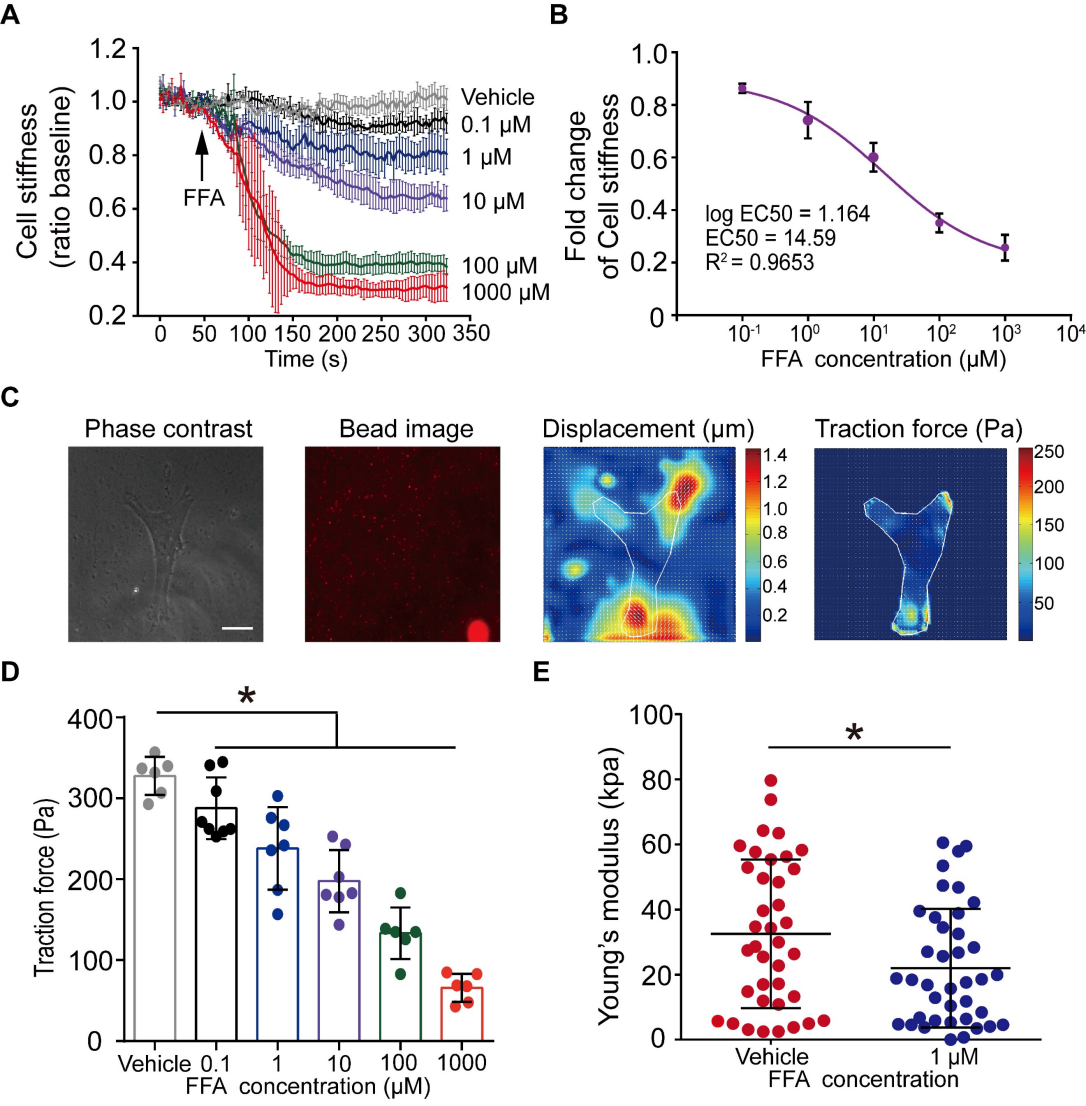
** Dose-dependent effect of flufenamic acid (FFA) on cell stiffness, traction force and Young's modulus of ASMCs.** (**A**) Time-courses of cell stiffness reduction of ASMCs before and after exposure to FFA at 0 (Vehicle), 0.1, 1, 10, 100, 1000 µM, respectively (n = 3). (**B**) Dose-response curves and EC50 values of FFA for fold change of cell stiffness in ASMCs (n = 3). (**C**) From left to right, phase-contrast image of the cell, fluorescent image of the microbeads, calculated microbeads displacement and traction force distribution, which were used for FTTFM to quantify the traction force of ASMCs (scale bar: 20 µm). (**D**) Quantified traction force of ASMCs in response to treatment with FFA for 5 min at concentration of 0 (Vehicle), 0.1, 1, 10, 100, 1000 µM, respectively (n = 6-8). (**E**) Young's modulus of ASMCs measured by AFM in the absence (Vehicle) or presence of 1 µM FFA treatment (n = 38 from 5-6 cells). * *p* < 0.05.

Given that FFA turned out to be the most potent to relax ASMCs, as manifested by maximal efficacy to decrease cell stiffness, among the 18 selected bitter compounds, we thereafter focused on the characterization of various effects of FFA on mechanical behaviors of ASMCs related to bronchodilation. First, we used cell stiffness assay with OMTC to characterize the dose-effect relationship of FFA to relax ASMCs with a concentration of FFA ranging from 0.1 to 1000 μM. The results shown in **Figure 2A** indicate that FFA significantly decreased cell stiffness of ASMCs in a dose-dependent manner. The results also showed that FFA could induce a modest degree of relaxation amounting to ∼20% at 1 μM, and the dose-response curve indicated that FFA could relax ASMCs by 50% at the concentration of 14.59 μM, or the EC50 value was 14.59 μM (**Figure 2B**).

Traction force measured by FTTFM using images of the cell (phase contrast) and the microbeads (fluorescence) indicated distinct patterns of microbeads displacement and the corresponding traction force distribution within the cell (**Figure 2C**). Quantitative analysis of traction force variation of ASMCs in response to FFA treatment confirmed that FFA incrementally decreased cell traction force to 287.69 ± 35.66 Pa (*p* = 0.043), 238.15 ± 47.34 Pa (*p* = 0.002), 197.72 ± 35.66 Pa (*p* = 0.001), 133.37 ± 29.13 Pa (*p* = 0.001) and 65.87 ± 15.90 Pa (*p* = 0.001) as compared to the control group (Vehicle, 327.59 ± 21.43 Pa) when the concentration of FFA increased from 0.1, 1, 10, 100, to 1000 µM, respectively (**Figure 2D**). Such dose-dependent response of traction force was consistent with that of cell stiffness.

Furthermore, we confirmed the efficacy of 1 µM FFA to relax ASMCs as characterized by the FFA-induced variation of Young's modulus of ASMCs using AFM. Compared with the control group (Vehicle) in 5 min, 1 μM FFA decreased the Young's modulus of ASMCs significantly (**Figure 2E**). Together, all these results show that FFA could rapidly relax ASMCs even at 1 µM, suggesting that FFA may have the potential to be used as a bronchodilator for the treatment of respiratory diseases such as asthma in a small dose.

### Flufenamic acid was as potent as conventional β-agonist to relax ASMCs cultured *in vitro* and abrogate airway resistance of asthmatic mice* in vivo*

**Figure 3:**
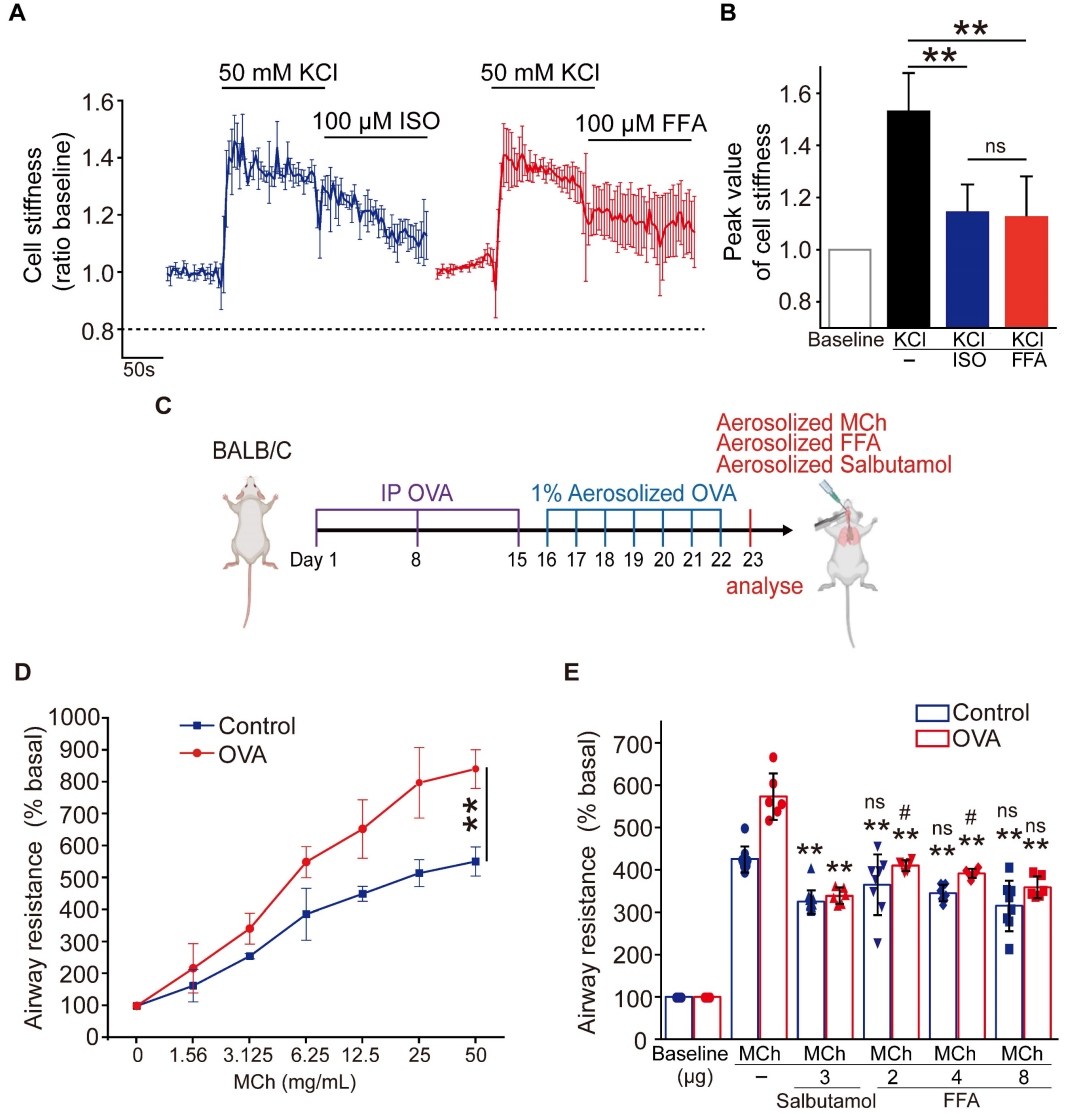
** Comparison of flufenamic acid (FFA, TAS2R14 agonist) and isoproterenol (ISO, β-agonist) for efficacy in reducing cell stiffness of ASMCs cultured in *vitro*, or FFA and salbutamol (β-agonist) for efficacy in reducing airway resistance of the OVA-induced BALB/c mouse model of asthma.** (**A**) Time-courses of cell stiffness variation (ratio baseline) of ASMCs in response to 50 mM KCl and 100 µM FFA or 100 µM ISO (n = 3). (**B**) Quantified peak value of cell stiffness induced by FFA or ISO after KCl (n = 3). (**C**) Schematic diagram of experimental protocol: preparing BALB/c mouse model of asthma by OVA-sensitization and challenge, analyzing the mouse model for asthma features, evaluating effects of MCh, FFA, salbutamol on airway resistance of the mouse model. (**D**) Airway resistance versus MCh concentration of the OVA-treated mice (OVA) and non-treated mice (Control) (n = 6-8 mice). (**E**) Airway resistance in percentage of the baseline value (% basal) of the OVA-treated mice (OVA) and the non-treated mice (Control) as measured before and after inhalation of MCh, salbutamol and FFA at different dosages (n = 6-12 mice, ** *p* < 0.01 versus corresponding MCh group; # *p* < 0.05, ns *p* > 0.01 versus corresponding MCh + 3 µg salbutamol group). A-D: ns *p* > 0.05, ** *p* < 0.01.

To further assess the potential of FFA as a bronchodilator to relieve asthma symptoms, we compared the efficacy of FFA in reducing cell stiffness of ASMCs to that of isoproterenol (ISO) which is a conventional β-agonist used to relax ASMCs. We first tested ISO's ability to reduce cell stiffness of ASMCs cultured *in vitro*. The cell stiffness reduction rate of ASMCs after being treated with ISO (100 μM) was about 30%, which was less than that with FFA (**Figure S1**). We also compared the ability of FFA and ISO to reduce cell stiffness of the ASMCs precontracted by KCl or methacholine (MCh). ASMCs cultured *in vitro* were pretreated with 50 mM KCl or 10 μM MCh to evoke cell contraction, and subsequently treated with 100 μM FFA or 100 μM ISO to relax the precontracted ASMCs. The results showed that 100 μM FFA and 100 μM ISO decreased the cell stiffness (ratio baseline) of precontracted ASMCs induced by KCl from 1.53 ± 0.05 to 1.12 ± 0.07 (*p* = 0.006) and 1.12 ± 0.04 (*p* = 0.004), respectively. The time-courses and averaged cell stiffness reduction elicited by FFA (100 μM) and ISO (100 μM) were similar (*p* = 0.973) (**Figure 3A/B**). In addition, the efficacy of FFA in reducing cell stiffness of the ASMCs precontracted by MCh (10 μM) was higher than that of ISO (*p* = 0.035) (**Figure S2**).

Then we compared the efficacy of FFA in reducing airway resistance of OVA-induced BALB/c mouse models of asthma, to that of salbutamol which is a conventional β-agonist-based bronchodilator used to relieve airway constriction of asthmatic patients. BALB/c mice were treated (sensitized and challenged) with OVA to induce asthmatic pathologies, and then stimulated with MCh to induce excessive airway constriction followed by treatment with FFA or salbutamol to relieve the airway constriction as illustrated in **Figure 3C**. The OVA-treated mice were first analyzed for features of asthma pathologies including airway remodeling, airway inflammation, and lung function, 24 hours after the final challenge. HE-stained lung tissues of OVA-treated mice showed enhanced smooth muscle layers around the bronchi as compared to the controls (**Figure S3A**). BALF samples of OVA-treated mice showed a dramatically increased proportion of eosinophils when compared to that of the controls** (Figure S3B)**. In addition, the OVA-treated mice exhibited a greater increase of airway resistance above the baseline level (% basal) when exposed to MCh as compared to the mice not treated with OVA, suggesting OVA-treated mice developed airway hyperresponsiveness (Control vs. OVA in **Figure 3D**). Taken together, it was verified that the OVA-treated mice presented cardinal pathological features of allergic asthma.

Next, we measured airway resistance of the OVA-treated mice that were challenged by MCh and subsequently treated with either FFA or salbutamol. As shown in **Figure 3E**, we first challenged the OVA-treated mice (OVA), and the non-treated mice (Control) with MCh at a concentration of 6.25 mg/mL, and 25 mg/mL, respectively, to induce more than 5-fold increase of airway resistance from the baseline value (% basal) in both groups. Afterwards the mice inhaled either aerosolized salbutamol (3 μg) or FFA (2, 4, and 8 μg) to relieve the 5-fold increase of airway resistance. We observed that inhalation of 3 μg salbutamol reduced airway resistance (% basal) by almost half in both OVA and Control group. In comparison, inhalation of 2-8 μg FFA also caused a dose-dependent reduction of airway resistance of mice in both OVA and Control groups. Specifically, inhalation of 8 μg FFA reduced airway resistance of the OVA-treated mice from 573.13 ± 49.78 to 358.64 ± 23.69 % (*p* = 0.001), and the non-treated mice from 425.42 ± 28.86 to 315.32 ± 55.04 % (*p* = 0.001), respectively, which was equally potent as 3 μg salbutamol (*p* = 0.147). Thus, these data suggest that FFA, a TAS2R14 agonist may have great potential to be used for bronchodilation in asthma therapy, as an alternative to the conventional β-agonists such as salbutamol.

### Flufenamic acid did rapidly increase [Ca^2+^]_i_ in ASMCs via TAS2R14 signaling pathway

**Figure 4:**
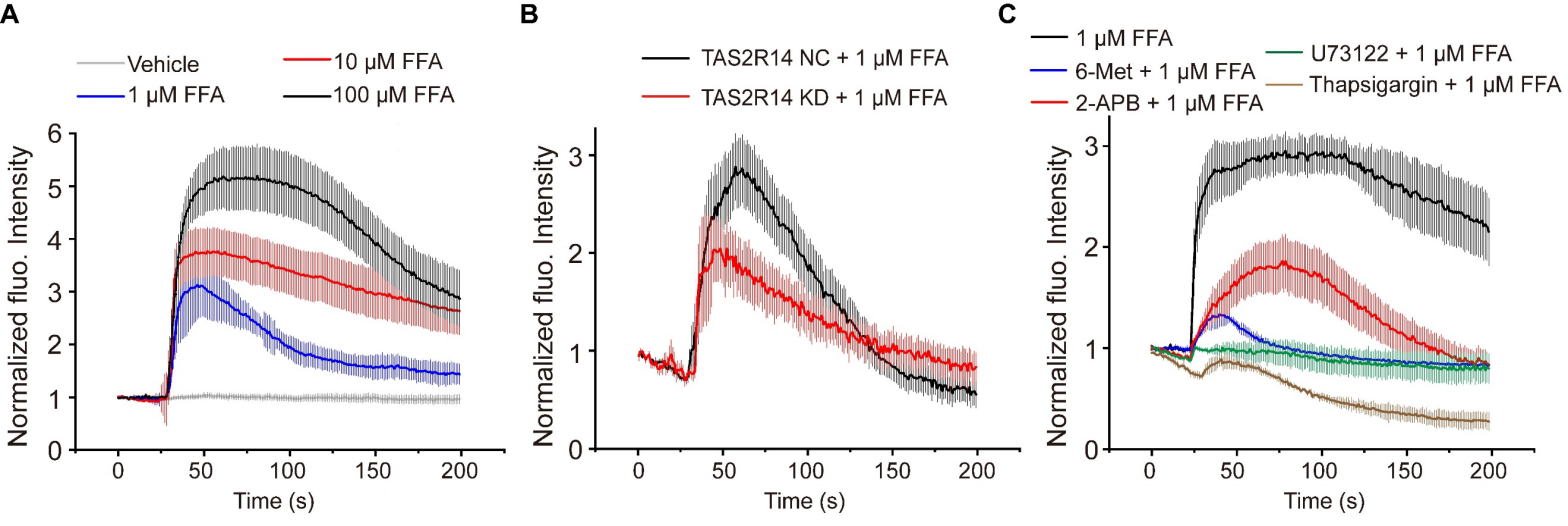
** FFA-induced rapid dose-dependent Ca^2+^ signaling in ASMCs and its mediation through activation of TAS2R14 and ER Ca^2+^.** (**A**) Time-courses of [Ca^2+^]_i_ level in cultured ASMCs before and after exposure to FFA at different dosage (1, 10, 100 µM, respectively). The [Ca^2+^]_i_ level was quantified by the fluorescence intensity of Fluo-4 labeled cells with exposure to FFA and normalized to the baseline intensity of the same cells without exposure to FFA (Vehicle). (**B**) Time-courses of 1 µM FFA-induced [Ca^2+^]_i_ level in cultured ASMCs with TAS2R14 mRNA expression at either normal or knock-down level of (TAS2R14-NC vs. TAS2R14-KD). (**C**) Time-courses of 1 µM FFA-induced [Ca^2+^]_i_ level in cultured ASMCs pretreated for 30 min with/out 6-Met (TAS2R inhibitor, 500 µM), U73122 (PLCβ inhibitor, 20 µM), 2-APB (IP_3_ receptor blocker, 100 µM), or thapsigargin (SERCA pump blocker, 10 µM), respectively. A-C: n = 3.

Ca^2+^ signaling is known to be evoked by the activation of TAS2Rs with bitter agonists, which plays an essential role in bitter agonist-initiated ASMC relaxation 8, 42. To ascertain whether Ca^2+^ signaling is involved in FFA-induced relaxation of ASMCs, we evaluated [Ca^2+^]_i_ of the ASMCs with Fluo-4/AM before and after exposure to FFA. **Figure 4A** shows the dynamic changes of the normalized fluorescence intensity in cultured ASMCs treated with FFA at different concentrations (1, 10, 100 µM). In all cases, the dynamic change of [Ca^2+^]_i_ reached peak value within ~ 10 s. As the concentration increased to 100 µM, the peak of [Ca^2+^]_i_ response reached approx. 5.5-fold of the baseline value. These data suggest that FFA rapidly increased the [Ca^2+^]_i_ of ASMCs via a dose-dependent manner.

To confirm the specificity of TAS2R14 (the only TAS2R for FFA) in mediating FFA-enhanced calcium signaling, we evaluated the FFA-induced [Ca^2+^]_i_ in ASMCs which the mRNA expression of TAS2R14 was silenced by using small interfering RNA (siRNA). The efficacy of siRNA1 was up to 70% confirmed by qPCR (**Figure S4**). We found that inhibition of TAS2R14 expression specifically inhibited 1 µM FFA-induced Ca^2+^ signaling response by more than 33% (**Figure 4B**). This verified that the FFA-induced [Ca^2+^]_i_ response in ASMCs was indeed mediated via TAS2R14 signaling pathway.

Canonically, TAS2R activation initiates the release of Ca^2+^ from the endoplasmic reticulum (ER) with a cascade reaction of G protein, PLCβ2, and IP_3_ 8. To test whether the FFA-induced [Ca^2+^]_i_ was facilitated through this pathway, we pretreated ASMCs with 6-Met (TAS2R inhibitor, 500 µM), U73122 (PLCβ inhibitor, 20 µM), 2-APB (IP_3_ receptor blocker, 100 µM), or thapsigargin (SERCA pump blocker, 10 µM) for 30 min before exposure to FFA (1 µM). As shown in **Figure 4C**, pretreatment with various inhibitors of TAS2R signaling pathway significantly abrogated the FFA-enhanced calcium signaling. All these results show that FFA increased the [Ca^2+^]_i_ of ASMCs through activation of TAS2R14, PLCβ, IP_3_, and release of Ca^2+^ from ER.

Furthermore, before the discovery of TAS2R-medicated relaxation of ASMCs, the classic mechanism of relaxation for ASMCs is thought to be mediated by increasing expression of cAMP 43, 44. To test whether FFA relaxed ASMCs via increasing cAMP, we measured the cAMP level inside ASMCs treated with FFA at 1, 10, 100, and 1000 µM, respectively, by using ELISA assay 16. As shown in **Figure S5**, we found that FFA did not change the cAMP level inside ASMCs regardless of concentration, suggesting that the FFA-induced ASMC relaxation was independent of cAMP but more likely to be mediated by TAS2R signaling pathway.

### Flufenamic acid relaxed ASMCs via TAS2R14 signaling and the opening of BK_Ca_ channels

**Figure 5:**
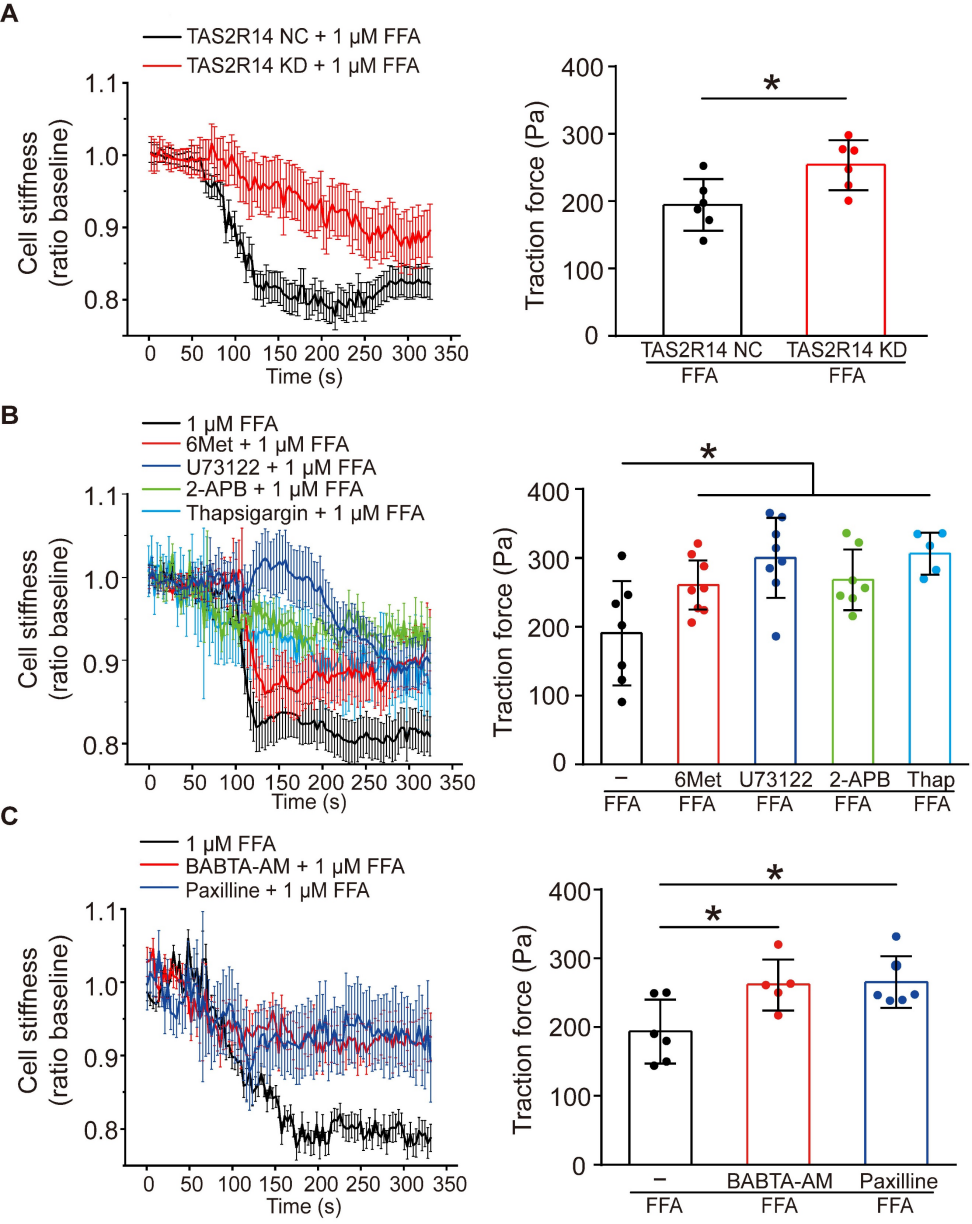
** Effects of TAS2R14 signaling and BK_Ca_ channels opening on FFA-induced reduction of cell stiffness and traction force in cultured ASMCs.** (**A**) Cell stiffness and traction force of cultured ASMCs pretreated with TAS2R14 KD siRNA or TAS2R14 NC was measured before and after exposure to 1 µM FFA. (**B**) Cell stiffness and traction force of cultured ASMCs pretreated with 6-Met, U73122, 2-APB or thapsigargin was measured before and after exposure to 1 µM FFA. (**C**) Cell stiffness and traction force of cultured ASMCs pretreated with BABTA-AM or paxilline was measured before and after exposure to 1 µM FFA. For cell stiffness, n = 3; for traction force, n = 6-8, * *p* < 0.05.

To confirm whether the FFA-induced reduction of cell stiffness and traction force of ASMCs were also mediated through the same signaling pathway of TAS2R14, PLCβ, IP_3_ activation and ER Ca^2+^ release, we first compared the reduction of cell stiffness and traction force induced by 1 µM FFA in cultured ASMCs with/out knockdown of TAS2R14 mRNA expression by siRNA. The results indicate that knockdown of TAS2R14 mRNA expression indeed significantly attenuated the 1 µM FFA-induced reduction of cell stiffness and traction force as compared to no knockdown (**Figure 5A**).

Next, we analyzed the effect of 6-Met, U73122, 2-APB, and thapsigargin on the FFA-induced reduction of cell stiffness and traction force in cultured ASMCs. As shown in **Figure 5B**, inhibition of TAS2R14 signaling pathway by pretreatment with these inhibitors did attenuate to various extents the reduction of cell stiffness and traction force in ASMCs in response to 1 µM FFA. BAPTA-AM (10 µM), a well-known membrane permeable Ca^2+^ chelator also significantly attenuated the FFA-induced reduction of cell stiffness and traction force in the cultured ASMCs (**Figure 5C**). These data indicate that FFA-induced relaxation of ASMCs may indeed be related to the release of Ca^2+^ from ER due to FFA-induced activation of TAS2R14, PLCβ, and IP_3_.

Furthermore, we reasoned that the FFA-decreased cell stiffness and traction force in ASMCs might also involve BK_Ca_ channels as in most other cases of drug-induced ASMC relaxation. To confirm the possible involvement of BK_Ca_ channels in the FFA response of ASMCs, we performed whole-cell patch clamping recordings of the currents of BK_Ca_ channels in ASMCs. Ionic channels in ASMCs were activated by depolarization voltage between -60 and +70 mV from a holding potential of -70 mV to elicit total outward K^+^ currents. The oscillatory current was inhibited by paxilline, a BK_Ca_ channel blocker (5 µM), indicating the presence of a typical BK_Ca_ current (**Figure S6 A/B**). Under identical voltage-clamp conditions, we observed that the ratio of peak current amplitude to the cell membrane capacitance (pA/pF) at 70 mV was larger in the FFA-treated cells than that in the counterpart control cells (Control vs. + FFA, *p* = 0.029). Furthermore, the FFA-enhanced current density at 70 mV was inhibited in the cells pretreated with paxilline (Paxilline + FFA vs. + FFA, *p* = 0.001, **Figure S6 C/D**). Accordingly, we still observed that inhibition of BK_Ca_ channels significantly abolished FFA-induced reduction of cell stiffness and traction force (**Figure 5C**). These findings suggest that FFA-induced relaxation of ASMCs may be medicated by not only TAS2R14 signaling but also BK_Ca_ channels.

Intriguingly, FFA may inhibit TMEM16A (a calcium-activated chloride channel, CaCC), which may confound the relaxatioin effect of FFA on ASMCs. To evaluate the potential contribution of CaCCs on the relaxation effect of FFA on ASMCs, we measured the 1 µM FFA-induced reduction of cell stiffness in ASMCs after the mRNA expression of TMEM16A was silenced by using siRNA. As shown in **Figure S7**, knockdown of TMEM16A mRNA expression did not attenuate the 1 µM FFA-induced reduction of cell stiffness in ASMCs as compared to the cells without knockdown of TMEM16A mRNA expression. This demonstrated that CaCCs or at least their associated TMEM16A signaling was not involved in the 1 µM FFA-induced relaxation of ASMCs.

### Flufenamic acid induced the relaxation and inhibited migration of ASMCs for the long-term treatment

**Figure 6:**
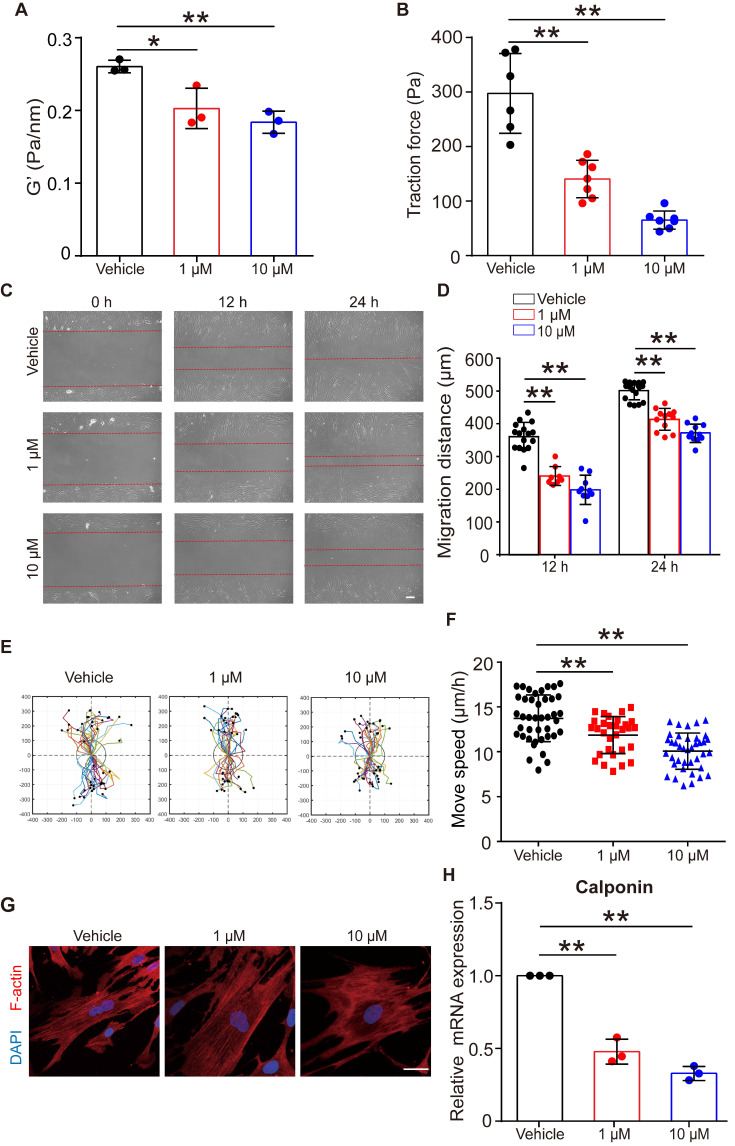
** Effects of long-term exposure to FFA on relaxation, migration, cytoskeleton reorganization and contractile protein expression of ASMCs.** (**A, B**) Cell stiffness (n = 3) and traction force (n = 6-7) of cultured ASMCs exposed to FFA at 0 (Vehicle), 1 and 10 μM, respectively, for 24 h. (**C**) Representative images of ASMCs migrating from cell-populated to cell-free regions at 0, 12 and 24 h after scratch wound (scale bar: 100 µm). (**D-F**) Quantified migration distance, displacement, and speed of ASMCs at 0, 12 and 24 h after scratch wound (n = 10-12 scenes, 30 cells, 20-30 cells, respectively). The displacement of each cell was tracked in the view field for 24 h, the trajectory was randomly colored and marked by a black dot to indicate its end-point and the total distance travelled by the cell. (**G**) Representative images of immunofluorescence labeled F-actin stress fibers (Red) and nucleus (Blue) in cultured ASMCs with 24 h exposure to FFA at 0 (Vehicle), 1, and 10 µM, respectively (scale bar: 10 µm). (**H**) Calponin mRNA expression in cultured AMSCs with 24 h exposure to FFA at 0 (Vehicle), 1, and 10 µM, respectively, as detected by qPCR (n = 3). * *p* < 0.05, ** *p* < 0.01.

Given that FFA relaxed ASMCs rapidly, we further analyzed whether it could maintain such effects on ASMCs during long-term exposure to FFA. First, we confirmed that long-term exposure to FFA (24 h, 1 µM and 10 µM) was not cytotoxic to ASMCs (**Figure S8**). Then, we measured cell stiffness and traction force of ASMCs pretreated with FFA for 24 h. We found that 24 h exposure of FFA maintained a significant reduction of cell stiffness and traction force of the cultured ASMCs (**Figure 6A/B**).

**Figure 6C** showed representative images of cell migration during a wound healing assay for ASMCs treated with FFA at 1 µM or 10 µM for 24 h. In all cases, cells always migrated from cell-populated to cell-free regions, but the FFA-treated cells appeared to migrate much less efficiently. Quantitatively, the migration distance of the two closing borderlines of the cell-populated area kept increasing with time (24 h vs. 12 h) in a time-dependent manner (**Figure 6D**). The migration of ASMCs also exhibited distinct patterns of increasingly reduced displacement scattering and speed with increasing dosage of FFA (**Figure 6E/F**).

Since contraction/relaxation as well as migration of ASMCs are all associated with reorganization of cytoskeletal stress fibers, we thus observed the F-actin stress fibers in ASMCs with/without exposure to FFA for 24 h. As shown in **Figure 6G**, ASMCs exposed to FFA exhibited increasingly hollow and slim stress fibers with increasing dosage of FFA (1 µM vs. 10 µM), as compared to the ASMCs without exposure to FFA (Vehicle). The mRNA expression of calponin, a major contractile protein in ASMCs also decreased by ~ 40% to ~70% when FFA increased form 1 µM to 10 µM, as compared to that without FFA (Vehicle), which is similar to the dose-dependent decrease observed in all the above assays (**Figure 6H**).

These data indicate that long-term exposure to FFA may keep ASMCs relaxed and inhibited from migration by decreasing calponin expression, suggesting the potential of FFA to meet additional requirements for being used as asthma controller medicine.

### Flufenamic acid also induced an anti-inflammatory response in cultured ASMCs

**Figure 7:**
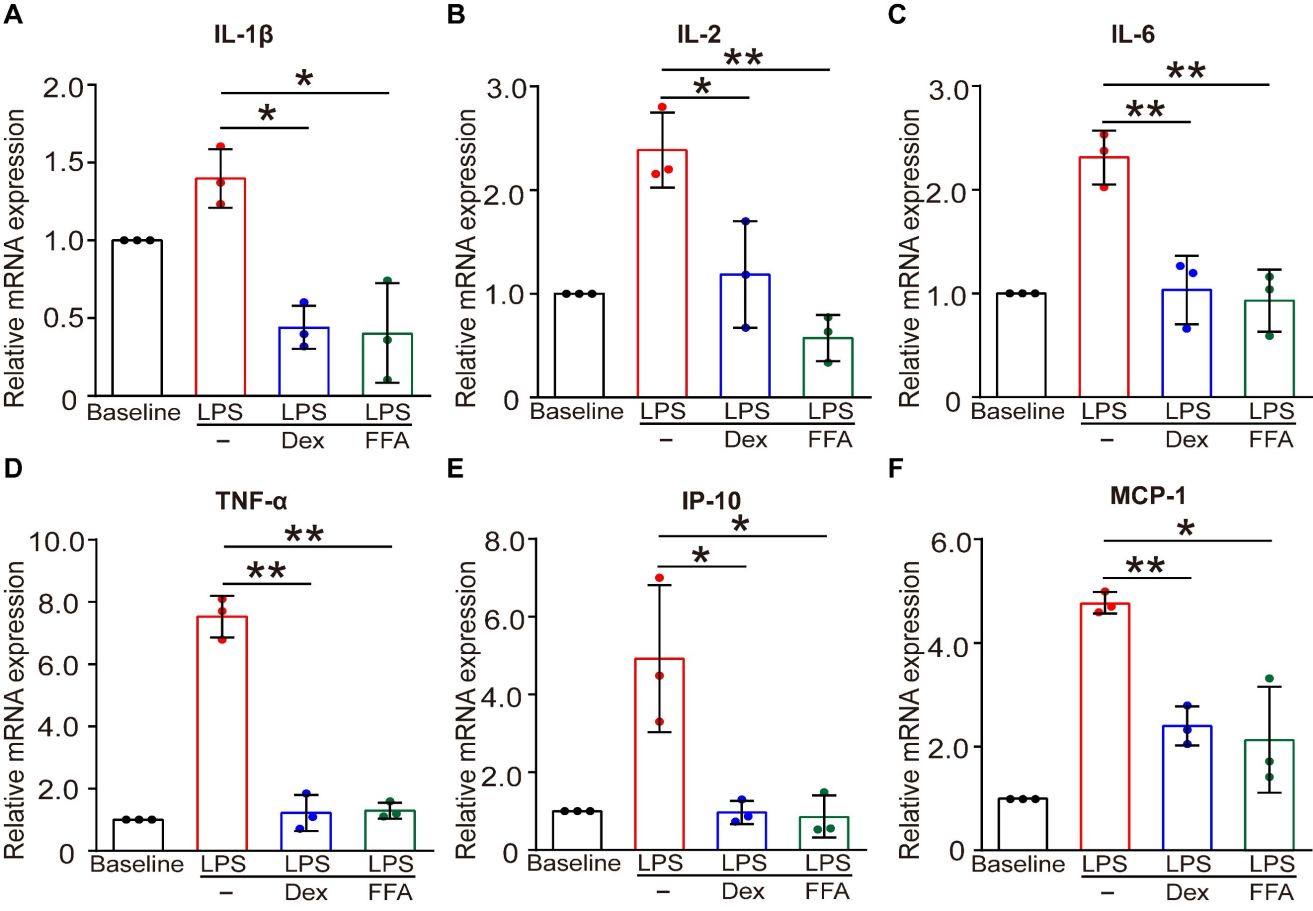
** Effects of FFA on LPS-induced release of inflammatory cytokines in cultured ASMCs.** (**A-F**) mRNA expression level of IL-1β, IL-2, IL-6, TNF-α, IP-10, and MCP-1 as detected by qPCR in ASMCs stimulated with LPS (1 µg/mL) in the absence or presence of 1 µM FFA or 1 µM Dex for 24 h. n = 3, LPS + FFA, LPS + Dex vs. LPS, * *p* < 0.05, ** *p* < 0.01.

In addition to the function of contraction/relaxation, ASMCs are also known to release inflammatory cytokines in the airway system during asthma pathogenesis and thus provide important targets for anti-inflammatory therapy 45. Considering that FFA is a well-known nonsteroidal anti-inflammatory drug (NSAID) already used in clinical practice for alleviating inflammation, it would offer additional benefit in asthma therapy if FFA could also suppress the inflammatory response of the ASMCs 46. Therefore, we investigated the effect of FFA on LPS-induced acute inflammatory response in ASMCs by measuring mRNA expression of key inflammatory cytokines associated with asthma using qPCR. The results showed that after exposure to LPS (1 µg/mL) for 24 h, mRNA expression levels of key inflammatory cytokines including IL-1β, IL-2, IL-6, IP-10, MCP-1, and TNF-α were all markedly elevated. However, these elevated levels of cytokines in ASMCs were all largely suppressed by the presence of 1 µM FFA in the culture medium, and the suppression by FFA was equally if not more effective than the treatment with 1 µM Dex, a conventional steroid widely used for treating inflammatory disease such as asthma (**Figure 7**). These results provided convincing evidence that FFA also targeted ASMCs to suppress cytokine release besides promoting airway relaxation, suggesting that FFA may well be a dual-functional drug for treating asthma.

## Discussion

In this study, we systematically retrieved and analyzed bitter compounds in BitterDB database followed by cell stiffness screening to find the most potent bitter compound as potential alternative bronchodilator based on TAS2R activation instead of the conventional but problematic β-agonists. We first retrieved the 1041 bitter compounds in BitterDB and then analyzed their respective subtypes of TAS2Rs as well as their effective concentrations. We found 18 bitter compounds that could activate the subtypes of TAS2Rs highly expressed in ASMCs (TAS2R5, 10, 14) with low effective concentrations.

We then found that among these bitter compounds, FFA was the most potent for relaxing ASMCs based on cell stiffness screening. We also verified that FFA mediated relaxation of ASMCs by activating TAS2R14 signaling pathway and opening BK_Ca_ channels, and the efficacy of FFA was similar to that of conventional β-agonist to relax ASMCs and abrogate airway resistance in animal models of asthma.

It is important to note that the combination of BitterDB database analysis and cell stiffness screening enabled us to identify FFA targeting TAS2R14 as the most potent bitter compound to relax ASMCs. From BitterDB database analysis, we were able to narrow down 18 out of 1041 existing compounds, which are known to activate the three subtypes of TAS2Rs (TAS2R5, 10, and 14) considered critical for mediating relaxation of ASMCs at low effective concentrations. Cell stiffness screening, however, revealed that these 18 compounds might well have different efficacy in relaxing ASMCs even if they have the same effective concentration for activating TAS2Rs. For example, BitterDB database analysis identified that pentagalloylglucose (TAS2R5 agonist) and mefenamic acid (TAS2R14 agonist) had the same effective concentration for TAS2R activation. But cell stiffness assay showed that pentagalloylglucose could dramatically increase cell stiffness of ASMCs, while in contrast mefenamic acid was able to decrease cell stiffness of ASMCs. Similarly, 1,10-phenanthroline (TAS2R5 agonist) and chloramphenicol (TAS2R10 agonist) with same effective concentration for TAS2R activation, but the former was more effective than the latter in relaxing ASMCs. Therefore, only combined results from both BitterDB database analysis and cell stiffness screening could lead to identification of FFA activating TAS2R14 as the most potent bitter compound for relaxing ASMCs. Interestingly, TAS2R14 has a large amount of agonist-selective contact points likely exceeding that of all other promiscuous TAS2Rs according to homology modeling, molecular docking, and point mutagenesis experiments 47. Therefore, it is perhaps not surprising that eventually the FFA targeting TAS2R14 turned out to be the most potent bitter agonist for relaxing ASMCs.

In addition to the potency of FFA in rapidly relaxing ASMCs, we also demonstrated that FFA could maintain its capacity to relax ASMCs during long-term exposure. This is essential for FFA as a bronchodilator in asthma therapy because it may be required not only for relieving acute airway constriction during asthma attacks, but also for long-term management of chronic asthma symptoms when taken regularly as prescribed asthma controller medicine. Currently prescribed asthma controller medications include inhaled corticosteroids, leukotriene modifiers, long-acting β-agonists, and immunomodulators, which work in different ways to ward off wheezing, coughing, shortness of breath, and other common symptoms 48-50. Among them, only long-acting β-agonists are bronchodilators, and their effects can last for over 12 h. The fact that FFA could maintain inhibitory effects on mechanical behaviors such as cell stiffness, traction force and migration of ASMCs for 24 h as revealed in our study suggests that FFA may have great potential to replace long-acting β-agonists as alternative asthma controller medicine.

Technically speaking, several bitter agonists have been screened for their potency of relaxing ASMCs by measuring traditional [Ca^2+^]_i_ release in the cells 51. However, this approach seems to be inefficient for screening a large number of compounds, let alone the fact that Ca^2+^ signaling may be involved in both relaxation and contraction of ASMCs 52, 53. In contrast, mechanical properties such as cell stiffness and traction force measured by OMTC, FTTFM, and AFM have been used to determine relaxation/contraction of ASMCs 26, 29, 54. In particular, the cell stiffness assay by OMTC provides a highly efficient method to evaluate and compare different compounds for their potencies in relaxing ASMCs at the collective cell level. Thus, in this study we adopted the cell stiffness assay by using OMTC to screen for the most potent for relaxing ASMCs from those 18 compounds resulting from BitterDB database analysis. Such cell stiffness screening successfully identified FFA as the most potent bitter compound to relax ASMCs, at least compared to the 1041 compounds in BitterDB database. Subsequent to cell stiffness screening, the efficacy of FFA to relax ASMCs was further corroborated by assessment of traction force, Young's modulus, cell migration, cytoskeleton (F-actin stress fibers) reorganization as well as contractile protein (calponin) expression, and all the evidence indicated that 1 μM FFA was sufficient to relax ASMC cultured *in vitro*. We also confirmed with both cell culture *in vitro* and OVA-induced mice *in vivo* that FFA was comparable to conventional β-agonist, ISO and salbutamol, respectively, in reducing stiffness of KCl/MCh-contracted ASMCs and airway resistance of MCh-challenged mice.

In this study, we attributed the mechanism of FFA-induced relaxation of ASMCs to [Ca^2+^]_i_ signaling with resultant opening of BK_Ca_ channels as Deshpande et al. proposed 8. However, the mechanism of bitter substance-induced relaxation of ASMCs following activation of TAS2Rs has not yet been fully elucidated. In fact, Zhang et al. found that patch-clamp recordings of smooth muscle cells show no activation of BK_Ca_ channels upon chloroquine treatment 55. And it was proposed that bitter substances reverse the increase in [Ca^2+^]_i_ evoked by bronchoconstrictors by suppression of L-type voltage-dependent calcium channels (VDCCs) 42. Recently, Zhou et al. Reported that bitter substances induced airway smooth muscle relaxation via mediating Gα_t_ release of TAS2R activation 56. Nevertheless, it has been shown in animal smooth muscle cells in coronary artery, portal vein and kidney that FFA activates BK_Ca_ channels detected by patch clamp recordings 57, 58. Therefore, we tested the effect of FFA on the activation of BK_Ca_ channels in our cultured ASMCs by path clamp experiment. Our results demonstrated that FFA could indeed open BK_Ca_ channels in ASMCs, and specific inhibition of BK_Ca_ channels partially inhibited the FFA-induced decrease of cell stiffness, thereby confirming to a certain extent that the BK_Ca_ channel was involved in the FFA-induced relaxation process of ASMCs.

Besides TAS2R14 activation, FFA is also known to inhibit of TMEM16A (a typical protein of CaCC) that also contributes to the relaxation of ASMCs 59, 60. CaCCs are present in various cell types including smooth muscle cells 61, 62. Several studies have indicated that TMEM16A positively regulates the contractile response of ASMCs both *in vitro* and *in vivo* 59, 63. And chemically antagonizing TMEM16A interrupts ion flux both at the plasma membrane and sarcoplasmic reticulum to acutely relax ASMCs 64. In this study, the knockdown of TMEM16A mRNA expression did not seem to affect 1 µM FFA-induced reduction of cell stiffness in the cultured ASMCs. These results suggest that the FFA-induced relaxation of ASMCs as we observed was not contributed to a large extent due to inhibition of TMEM16A associated with CaCCs, even though other mechanisms may not be ruled out completely.

In addition to bronchodilation, ASMCs are also recognized as attractive anti-inflammatory targets for treating chronic asthma because they are known to release cytokines that play a key role in orchestrating and perpetuating airway inflammation 65. For example, OVA-treated mice exhibit increased level of inflammatory cytokines including IL-4, IL-6 and TNF-α in the BALF, which can be inhibited by anti-inflammatory mahuang decoction, and patients with severe asthma have recently been approved to be treated by blocking antibodies against specific cytokines 66, 67. Consistent with these findings, our results demonstrated that FFA significantly attenuated the mRNA expression of inflammatory cytokines released from LPS-stimulated ASMCs, including IL-1β, IL-2, IL-6, IP-10, MCP-1, and TNF-α. Although the mechanism still needs to be carefully studied, these results suggest that FFA may be more than a single, but a dual-functional drug for controlling both airway hyperresponsiveness and inflammation in asthma therapy.

However, being a NSAID, FFA may potentially stimulate the production of pro-inflammatory factors such as leukotriene through inhibition of cyclooxygenase (COX), as has been observed for other NSAIDs 68, 69. In this study, we found little change in the level of leukotriene β4 in ASMCs when the cells were treated with FFA at 100 µM (data not shown). This suggests that the risk of FFA to cause inflammatory response in ASMCs may be minimal, especially when it is used at 1 µM to efficiently relax ASMCs. Furthermore, FFA is known to have various derivatives, and it has been shown that FFA analogs containing pentafluorosulfanyl potently activated ion channels while displayed little or no inhibitory effect on COX-1&2 at 100 μM 70. Therefore, it is highly possible that we can eventually design some FFA derivatives that can potently relax ASMCs but not stimulate production of inflammatory factors such as leukotrienes. Thus, we believe that FFA or its further developed derivatives are promissing alternatives for anti-asthma treatment, even though their efficacy and safety need to be thoroughly evaluated before clinical applications.

Finally, it is worth noting that BitterDB database was last updated in 2019, suggesting that our study may not cover the latest development of the database collection. In addition, our study only focused on the effect of FFA on features of allergic asthma model, yet it is necessary to study the effect of FFA on prophylactic asthma models. Furthermore, the current identification of FFA as the most potent bitter compound to relax ASMCs does not place us in a position to assume that FFA is necessarily a useful bronchodilator for treating asthma in clinic practice. This is because that there are plenty of FFA derivatives (such as FFA-d4) from which more potent compounds for relaxing ASMCs may be explored by establishing their structure-activity relationships in future, and any of these potential drugs will also require additional preclinical studies, including pharmacodynamics and pharmacokinetics, toxicology, and adverse effect profile before it can be approved for clinic treatment.

## Conclusion

We identified from BitterDB database analysis and cell stiffness screening that FFA was most potent to rapidly relax ASMCs via TAS2R14 signaling pathway in association with release of ER Ca^2+^ and opening of BK_Ca_ channels. The efficacy of FFA in relaxing ASMCs was comparable to that of conventional β-agonist both *in vitro* and *in vivo* in terms of drug-induced reduction of cell stiffness and airway resistance. FFA also inhibited migration and LPS-induced inflammatory response of ASMCs during long-term exposure. Taken together these data suggest that FFA might be a favorable drug agent for further development of TAS2R-based novel dual-functional medication of bronchodilation and anti-inflammation to treat respiratory disease such as asthma.

## Supplementary Material

Supplementary figures and table.

## Figures and Tables

**Table 1:** Bitter compounds subjected to cell stiffness screening.

No.	Name	CAS	Effective concentration (μM)	Activated TAS2Rs
1	Pentagalloylglucose	14937-32-7	3	TAS2R5
2	1,10-phenanthroline	66-71-7	100	TAS2R5
3	Cucurbitacin E	18444-66-1	0.01	TAS2R10
4	Denatonium benzoate	3734-33-6	3	TAS2R10
5	Quinine	130-95-0	10	TAS2R10
6	Benzoin	119-53-9	30	TAS2R10
7	Chloramphenicol	56-75-7	100	TAS2R10
8	Flufenamic Acid	530-78-9	0.01	TAS2R14
9	Chlorhexidine	55-56-1	0.1	TAS2R14
10	Tangeretin	481-53-8	0.3	TAS2R14
11	Kaempferol	520-18-3	0.5	TAS2R14
12	Quercetin	117-39-5	1	TAS2R14
13	Clonixin	17737-65-4	2	TAS2R14
14	Mefenamic acid	61-68-7	3	TAS2R14
15	Genistein	446-72-0	4	TAS2R14
16	Niflumic acid	4394-00-7	5	TAS2R14
17	Apigenin	461015-54-3	8	TAS2R14
18	Naringenin	480-41-1	10	TAS2R14
