# Supplementary material for: BitterDB database analysis plus cell stiffness screening identify flufenamic acid as the most potent TAS2R14-based relaxant of airway smooth muscle cells for therapeutic bronchodilation
Source: Theranostics. 2024 Feb 17;14(4):1744–63. doi: 10.7150/thno.92492 (PMC10879871; doi:10.7150/thno.92492)
Supplement: Supplementary file 1 — Supplementary figures and table. [file thnov14p1744s1.pdf]

BitterDB database analysis plus cell stiffness screening identify flufenamic acid as the most potent TAS2R14-based relaxant of airway smooth muscle cells for therapeutic bronchodilation

Kai Ni[1], Bo Che[1], Rong Gu, Chunhong Wang, Hongyang Xu, Huiduo Li, Shiyan Cen, Mingzhi Luo*, Linhong Deng*

Changzhou Key Laboratory of Respiratory Medical Engineering, Institute of Biomedical Engineering and Health Sciences, and School of Medical and Health Engineering, Changzhou University, Changzhou, Jiangsu, China

[1] These authors contributed equally to this work

Correspondence to:

Mingzhi Luo, +86-13616119565, luomingzhi@cczu.edu.cn;

Linhong Deng, +86-13685207009, dlh@cczu.edu.cn;

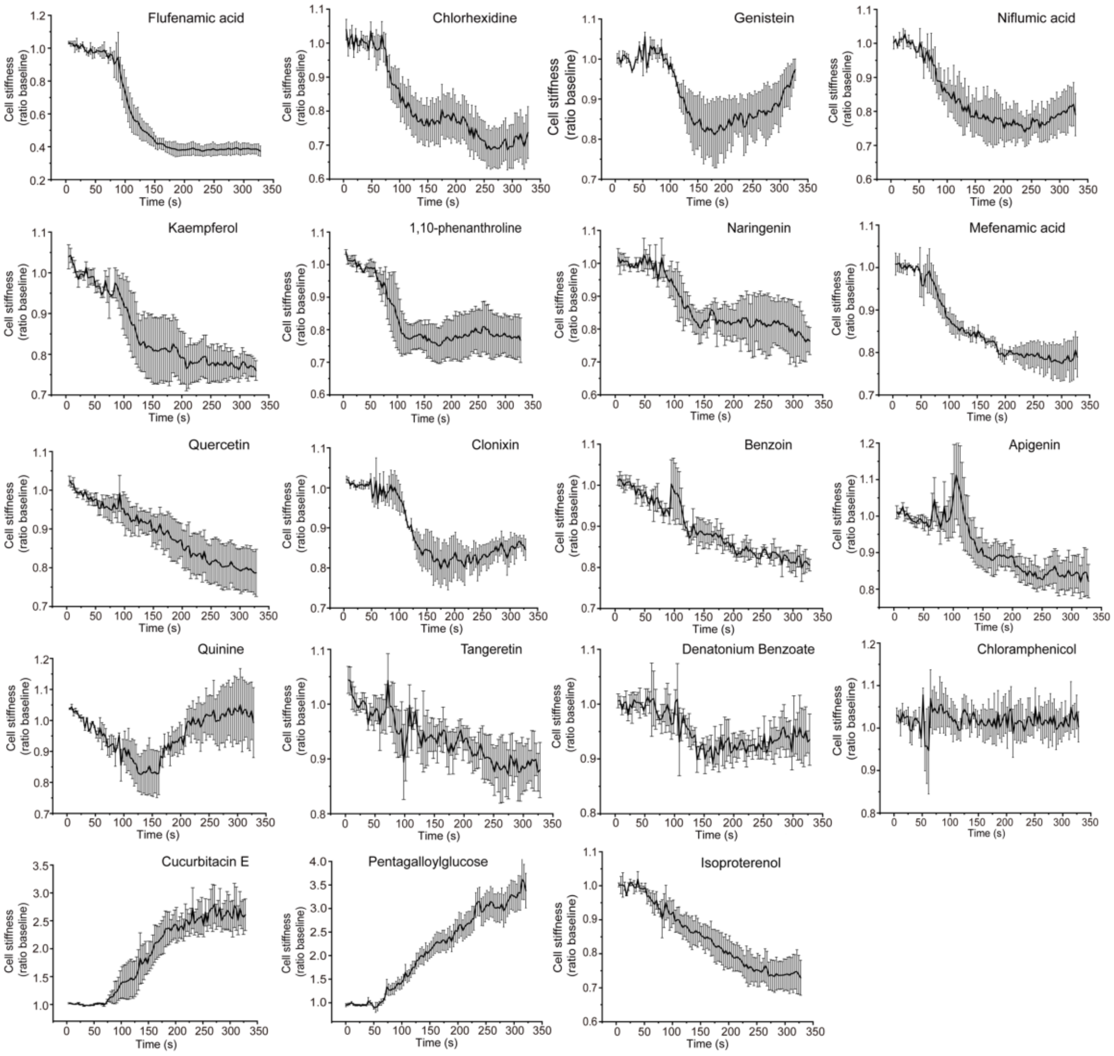

Figure S1. The dynamic changes in cell stiffness of ASMCs *in vitro* induced by 18 bitter agonists (100 μM) from Table 1 and isoproterenol (ISO, 100 μM) by using OMTC. Compounds were added to the culture medium at the 60 s time point. Data were shown as means ± SD, n = 3.

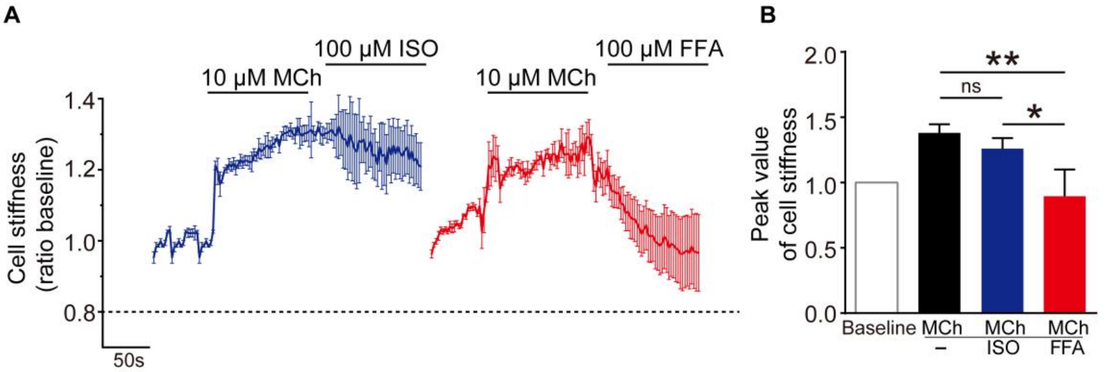

Figure S2. Effect of flufenamic acid (FFA, TAS2R14 agonist) and isoproterenol (ISO, β-agonist) on cell stiffness of *in vitro* cultured ASMCs pre-contracted with methacholine (MCh). (**A**) Time-courses of cell stiffness variation (ratio baseline) of ASMCs in response to 10 μM MCh and 100 μM ISO or 100 μM FFA. (**B**) Quantified peak value of cell stiffness induced by FFA or ISO after MCh. Data were shown as means ± SD, n = 3, ns indicated $p > 0.05$, * $p < 0.05$, ** $p < 0.01$.

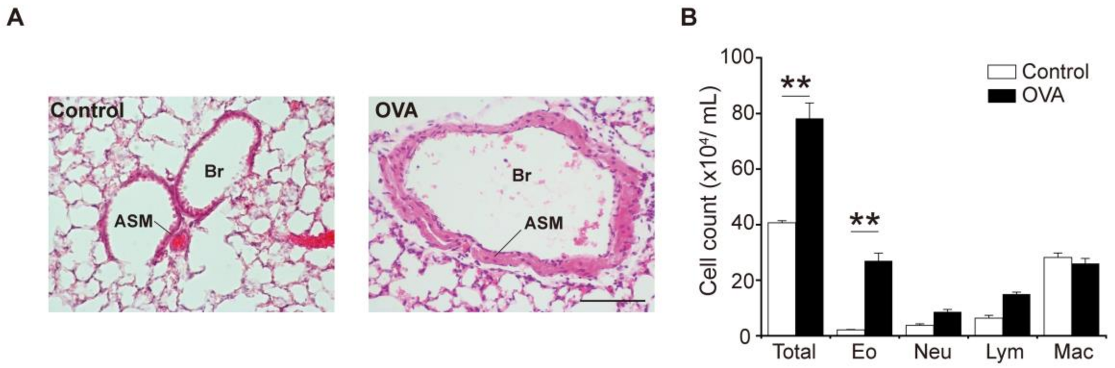

Figure S3. Pathological characterization of OVA-treated mice. (**A**) Representative photomicrographs of lung sections from BALB/c mice either not (Control), or OVA-sensitized and challenged (OVA). Br: bronchiole, ASM: airway smooth muscle, scale bar: 100 μm. (**B**) Total and differential cell counts of BALF in Control and OVA mice. Data were shown as means ± SD, n = 6-8 mice, ** $p < 0.01$.

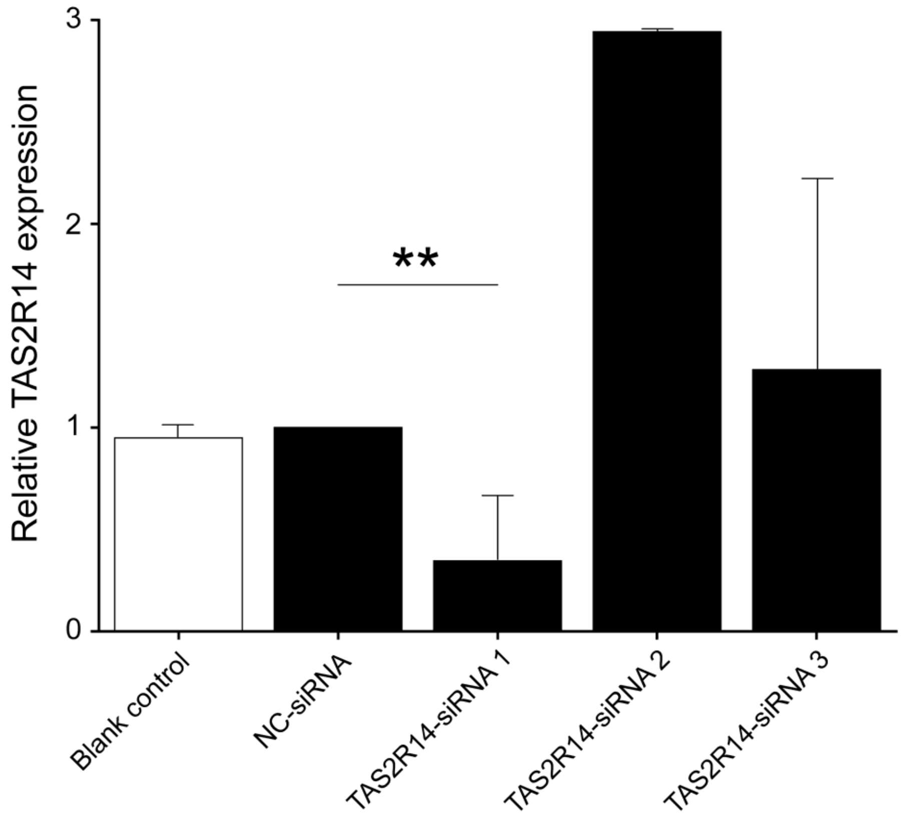

Figure S4. TAS2R14 mRNA expression in cultured ASMCs treated with TAS2R14 siRNA. Data were shown as means ± SD, n = 3, ** $p < 0.01$.

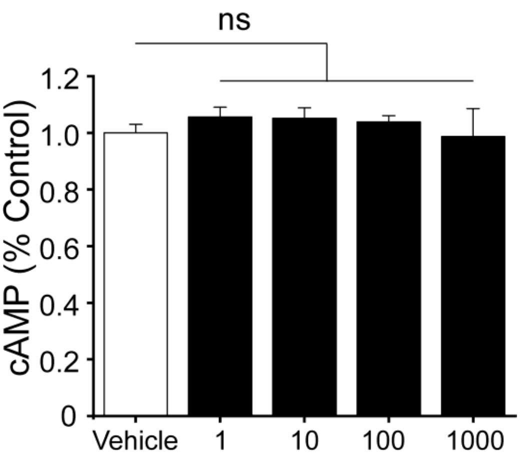

Figure S5. The cAMP level inside ASMCs treated with 0 (Vehicle), 1, 10, 100 and 1000 μM FFA for 30 min. Data were shown as means ± SD, n = 3, ns indicates $p > 0.05$.

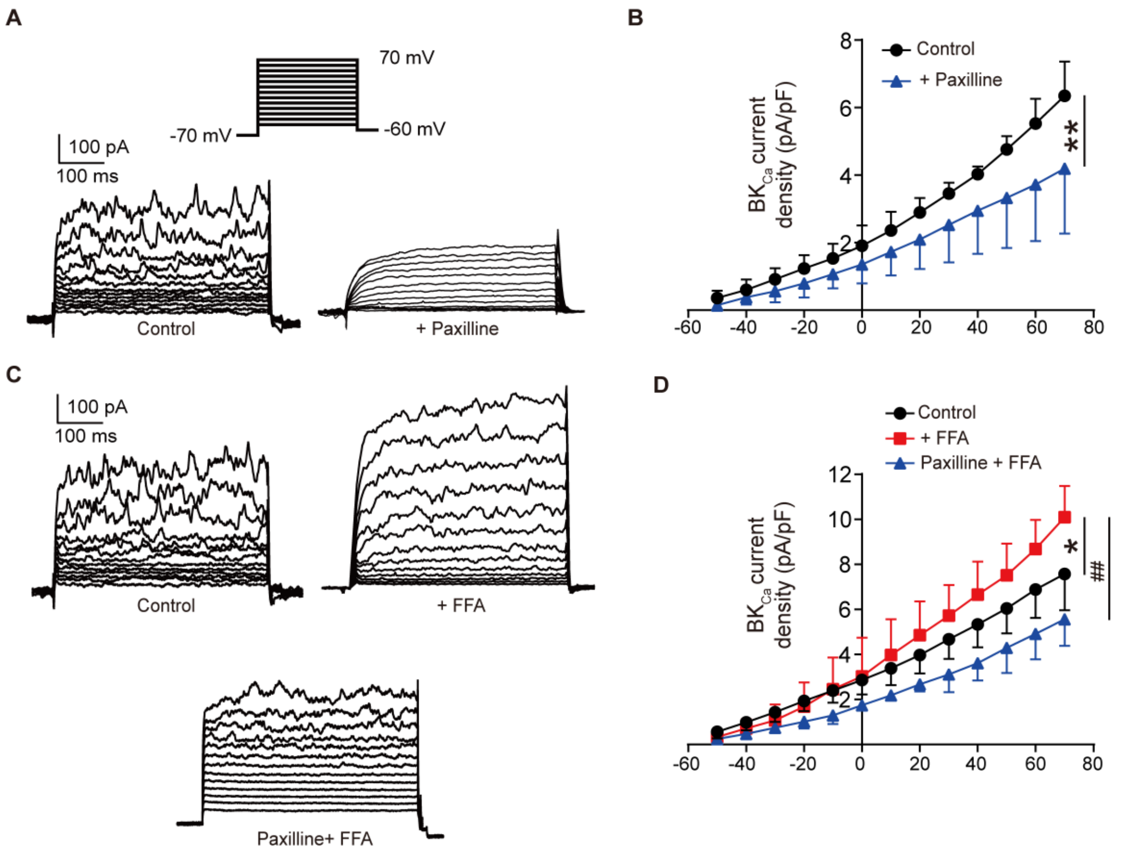

Figure S6. Whole-cell current of BK_{Ca} channels in ASMCs. (**A**) Outward currents were generated by depolarizing stimulation from -60 to 70 mV. The holding potential was -70 mV. Representative potassium currents recorded in ASMCs in the presence or absence of 5 μM paxilline. (**B**) *I-V* relationships of BK_{Ca} current density obtained in the same conditions as in (**A**) (n = 5). (**C**) Representative potassium currents recorded in ASMCs in the presence or absence of 1 μM FFA and representative potassium currents in ASMCs pretreated with paxilline as measured after exposure to 1 μM FFA. (**D**) *I-V* relationships of BK_{Ca} current density in ASMCs obtained in the same conditions as in (**C**) (n = 5). Data were shown as means ± SD, * $p < 0.05$, ** $p < 0.01$, ## $p < 0.01$.

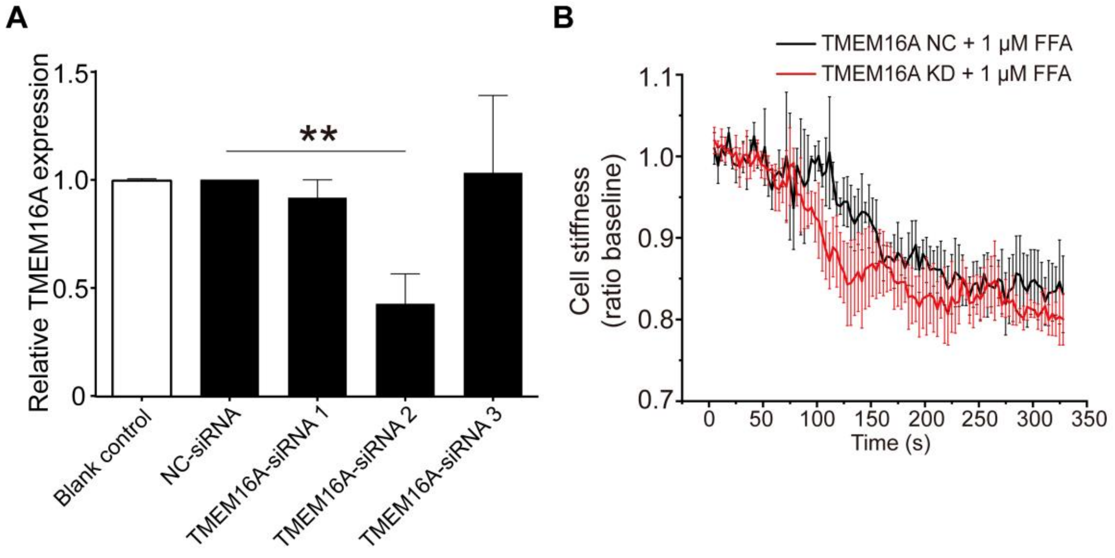

Figure S7. Effects of TMEM16A signaling on FFA-induced reduction of cell stiffness in cultured ASMCs. (**A**) TMEM16A mRNA expression in cultured ASMCs treated with TMEM16A siRNA. (**B**) Cell stiffness of cultured ASMCs pretreated with TMEM16A KD siRNA or TMEM16A NC, measured before and after exposure to 1 μM FFA. Data were shown as means ± SD, n = 3, ** $p < 0.01$.

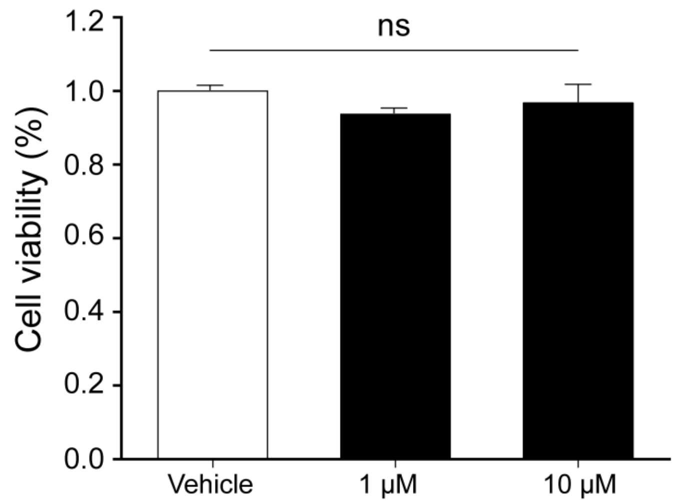

Figure S8. CCK-8 assay of cellular activity of ASMCs treated with or without FFA (1, 10 μM), respectively. Data were shown as means ± SD, n = 3, ns indicated $p > 0.05$.

Table S1 Compounds that activate TAS2R5, 10 and 14 in the BitterDB database

No.	Name	CAS	Effective concentration (μM)/EC50	Activated TAS2Rs	No.	Name	CAS	Effective concentration (μM)/EC50	Activated TAS2Rs
1	Pentagalloylglucose	14937-32-7	3	TAS2R5	14	Chlorpheniramine	113-92-8	10	TAS2R10
2	1,10-phenanthroline	66-71-7	100	TAS2R5	15	Dextromethorpha	125-71-3	10	TAS2R10
3	(-)-Epicatechin	490-46-0	1000	TAS2R5	16	Benzoin	119-53-9	30	TAS2R10
4	Procyanidin C2	37064-31-6	30000	TAS2R5	17	Parthenolide	20554-84-1	30	TAS2R10
5	Denatonium saccharide	90823-38-4		TAS2R5	18	Diphenidol	150969-57-6	30	TAS2R10
6	Sucralose	56038-13-2		TAS2R5	19	Haloperidol	337376-15-5	30	TAS2R10
7	Cucurbitacin E	18444-66-1	0.01	TAS2R10	20	Chloramphenicol	56-75-7	100	TAS2R10
8	Cucurbitacin B	6199-67-3	0.01	TAS2R10	21	Arborescin	6831-14-7	100	TAS2R10
9	Denatonium benzoate	3734-33-6	3	TAS2R10	22	Arglabin	84692-91-1	100	TAS2R10
10	Artemorin	64845-92-7	3	TAS2R10	23	Cascarillin	10118-56-6	100	TAS2R10
11	Strychnine	57-24-9	3	TAS2R10	24	Absinthin	1362-42-1	100	TAS2R10
12	Quinine	130-95-0	10	TAS2R10	25	(-)-alpha Thujone	546-80-5	100	TAS2R10
13	Papaverine	58-74-2	10	TAS2R10	26	Brucine	357-57-3	100	TAS2R10

27	Dapsone	14548-46-0	100	TAS2R10	43	Costunolide	553-21-9		TAS2R10
28	Cycloheximid	66-81-9	100	TAS2R10	44	Homoserine lactone	147852-83-3		TAS2R10
29	Methoxsalen	298-81-7	100	TAS2R10	45	Sparteine	90-39-1		TAS2R10
30	α-Santonin (-)-Form	481-06-1	100	TAS2R10	46	Atropine	51-55-8		TAS2R10
31	Caffeine	21399	300	TAS2R10	47	Cucurbitacin D	3877-86-9		TAS2R10
32	Coumarin	103802-83-1	300	TAS2R10	48	Cucurbitacin I	117793		TAS2R10
33	Quassin	76-78-8	300	TAS2R10	49	Tatridin A	41653-75-2		TAS2R10
34	Azathioprine	446-86-6	300	TAS2R10	50	Flufenamic Acid	530-78-9	0.01	TAS2R14
35	Yohimbine	146-48-5	300	TAS2R10	51	Chlorhexidine	55-56-1	0.1	TAS2R14
36	Erythromycin	114-07-8	300	TAS2R10	52	Tangeretin	481-53-8	0.3	TAS2R14
37	Famotidine	698387-09-6	300	TAS2R10	53	Cis-isohumulone		0.3	TAS2R14
38	D-camphor	464-49-3	300	TAS2R10	54	8-Isopentenylnaringenin	68682-02-0	0.3	TAS2R14
39	Picrotoxinin	17617-45-7	1000	TAS2R10	55	Kaempferol	520-18-3	0.5	TAS2R14
40	Bergapten	484-20-8	2800	TAS2R10	56	3,5-Diiodosalicylic Acid	133-91-5	0.5	TAS2R14
41	Chloroquine	19851	10000	TAS2R10	57	Quercetin	117-39-5	1	TAS2R14
42	Isopimpinellin	482-27-9	12000	TAS2R10	58	3,7,4-trihydroxyflavone	2034-65-3	1	TAS2R14

59	Trans-isohumulone	467-72-1	1	TAS2R14	75	(-)-alpha Thujone	546-80-5	3	TAS2R14
60	Trans-isocohumulone		1	TAS2R14	76	D-camphor	464-49-3	3	TAS2R14
61	Cis-isoadhumulone		1	TAS2R14	77	Lupulon	468-28-0	3	TAS2R14
62	Trans-isoadhumulone		1	TAS2R14	78	1-Naphthoic acid	86-55-5	3	TAS2R14
63	Cis-isocohumulone	68127-23-1	1	TAS2R14	79	Adlupulone	31769-60-5	3	TAS2R14
64	Aristolochic acid	10190-99-5	1.9	TAS2R14	80	Genistein	446-72-0	4	TAS2R14
65	Clonixin	17737-65-4	2	TAS2R14	81	6-methoxyluteolinb	520-11-6	4	TAS2R14
66	Datiscetinb	480-15-9	2	TAS2R14	82	Niflumic acid	4394-00-7	5	TAS2R14
67	Luteolin	491-70-3	2	TAS2R14	83	Pemirolast	69372-19-6	5	TAS2R14
68	Nobiletin	10236-47-2	2.41	TAS2R14	84	Apigenin	461015-54-3	8	TAS2R14
69	Mefenamic acid	61-68-7	3	TAS2R14	85	Flavone	525-82-6	8	TAS2R14
70	Artemorin	64845-92-7	3	TAS2R14	86	5,7,2'-trihydroxyflavone	73046-40-9	8	TAS2R14
71	Parthenolide	20554-84-1	3	TAS2R14	87	Morin	11128-85-1	8	TAS2R14
72	Isoxanthohumol	521-48-2	3	TAS2R14	88	3,6,3,4-tetrahydroxyflavone		8	TAS2R14
73	Xanthohumol	569-83-5	3	TAS2R14	89	Pinocembrin	480-39-7	8	TAS2R14
74	Picrotoxinin	17617-45-7	3	TAS2R14	90	Silibinind		8	TAS2R14

91	3,2-dihydroxychalcone	36574-83-1	8	TAS2R14	107	4-hydroxychalcone	38239-52-0	16	TAS2R14
92	2,2,4-trihydroxychalcone	26962-50-5	8	TAS2R14	108	Isoliquiritigenin	961-29-5	16	TAS2R14
93	Scutellarein	529-53-3	8	TAS2R14	109	Phloretinb	60-82-2	16	TAS2R14
94	(+/-) Equol		8	TAS2R14	110	Resveratrol	501-36-0	16	TAS2R14
95	Naringenin	480-41-1	10	TAS2R14	111	Sulfuretinc		16	TAS2R14
96	Quinine	130-95-0	10	TAS2R14	112	Diclofenac	15307-86-5	25	TAS2R14
97	Chloroquine	54-05-7	10	TAS2R14	113	Diphenhydramine	58-73-1	30	TAS2R14
98	Benzoin	119-53-9	10	TAS2R14	114	Haloperidol	337376-15-5	30	TAS2R14
99	Noscapine	128-62-1	10	TAS2R14	115	1,8-naphthaladehyde acid		30	TAS2R14
100	Papaverine	58-74-2	10	TAS2R14	116	Picrotin		30	TAS2R14
101	Diphenidol	150969-57-6	10	TAS2R14	117	Flavanone	487-26-3	32	TAS2R14
102	Hesperetin	520-33-2	16	TAS2R14	118	Eriodictyolb		32	TAS2R14
103	7,4-'dihydroxyflavone	2196-14-7	16	TAS2R14	119	Homoeriodictyolc	446-71-9	32	TAS2R14
104	5,7'-dimethoxyflavonee	21392-57-4	16	TAS2R14	120	Liquiritigenin	578-86-9	32	TAS2R14
105	7,3',4'-trihydroxyflavone	2150-11-0	16	TAS2R14	121	Chalcone	614-47-1	32	TAS2R14
106	Butein	21849-70-7	16	TAS2R14	122	Eriodictyolchalconec		32	TAS2R14

123	7-hydroxyisoflavoneg	13057-72-2	32	TAS2R14	139	Chlorpheniramine	113-92-8	100	TAS2R14
124	Miconazole	22916-47-8	33	TAS2R14	140	Methoxsalen	298-81-7	100	TAS2R14
125	Malathion	11096-67-6	50	TAS2R14	141	α-Santonin (-)-Form	481-06-1	100	TAS2R14
126	Dulcoside A	64432-06-0	50	TAS2R14	142	Piperonylic acid	94-53-1	100	TAS2R14
127	Chrysin	480-40-0	63	TAS2R14	143	Pantoprazole	02625-70-7	100	TAS2R14
128	(+)-taxifolin	20254-28-8	63	TAS2R14	144	Salsalate	552-94-3	100	TAS2R14
129	Pelargoninidin chloride		63	TAS2R14	145	Trp-Trp-Trp		100	TAS2R14
130	Biochanin Ag	491-80-5	63	TAS2R14	146	6,4'-dihydroxyflavone	63046-09-3	125	TAS2R14
131	7,8,4-trihydroxyisoflavone	75187-63-2	63	TAS2R14	147	6,7'-dimethoxyflavone	26964-27-2	125	TAS2R14
132	Absinthin	1362-42-1	100	TAS2R14	148	4'-hydroxy-6-methoxyflavone	4002-52-2	125	TAS2R14
133	Arborescin	6831-14-7	100	TAS2R14	149	Herbacetin	527-95-7	125	TAS2R14
134	Arglabin	84692-91-1	100	TAS2R14	150	Isorhamnetin	480-19-3	125	TAS2R14
135	Cascarillin	10118-56-6	100	TAS2R14	151	4,2,5-trihydroxychalconee		125	TAS2R14
136	Cucurbitacin B	6199-67-3	100	TAS2R14	152	5,4'-dihydroxyflavone	6665-67-4	250	TAS2R14
137	Falcarindiol	55297-87-5	100	TAS2R14	153	4'-hydroxy-7-methoxyflavone	32272-23-4	250	TAS2R14
138	Carisop	78-44-4	100	TAS2R14	154	5,7',4'-trimethoxyflavone	5631-70-9	250	TAS2R14

155	Myricetind	529-44-2	250	TAS2R14	171	Rebaudioside C	63550-99-2	400	TAS2R14
156	Quercetagetin	90-18-6	250	TAS2R14	172	Rubusoside	64849-39-4	400	TAS2R14
157	Cyanidin chloridec	13306-05-3	250	TAS2R14	173	Chrysoeriol	491-71-4	500	TAS2R14
158	Isoflavone	574-12-9	250	TAS2R14	174	Fustin	20725-03-5	500	TAS2R14
159	6,7,4-trihydroxyisoflavone	17817-31-1	250	TAS2R14	175	7,4-dimethoxyisoflavone	1157-39-7	500	TAS2R14
160	7,3,4-trihydroxyisoflavone	485-63-2	250	TAS2R14	176	Formomonetin	485-72-3	500	TAS2R14
161	Xanthonee	90-47-1	250	TAS2R14	177	Glyciteing	40957-83-3	500	TAS2R14
162	Hg-12		250	TAS2R14	178	Umbelliferones	1076-38-6	600	TAS2R14
163	Aglycon H.g.-12		250	TAS2R14	179	Stevioside	57817-89-7	600	TAS2R14
164	Coumestrol	479-13-0	250	TAS2R14	180	Rebaudioside A	58543-16-1	600	TAS2R14
165	Caffeine	21399	300	TAS2R14	181	Theobromine	83-67-0	1000	TAS2R14
166	Coumarin	103802-83-1	300	TAS2R14	182	Rebaudioside B	58543-17-2	1000	TAS2R14
167	Quassin	76-78-8	300	TAS2R14	183	Diphenylthiourea	102-08-9	1700	TAS2R14
168	Benzamide	1613-76-9	300	TAS2R14	184	Divinyl sulfoxide	1115-15-7	3000	TAS2R14
169	Sodium Benzoate	532-32-1	300	TAS2R14	185	Isopimpinellin	482-27-9	11100	TAS2R14
170	4-hydroxyanisol	150-76-5	300	TAS2R14	186	Skimmianine	5255-76-5	15800	TAS2R14

187	Herbolide D		TAS2R14	194	5-propyl-2-thiouracil	2954-52-1	TAS2R14
188	Glimepiride	93479-97-1	TAS2R14	195	Androsterone	53-41-8	TAS2R14
189	Costunolide	553-21-9	TAS2R14	196	Lidocaine	137-58-6	TAS2R14
190	Docosahexaenoic Acid	25377-50-8	TAS2R14	197	Pantothenic acid	79-83-4	TAS2R14
191	Homoserine lactone	147795-39-9	TAS2R14	198	Pyrocatechin	120-80-9	TAS2R14
192	Salicylic acid	29656-58-4	TAS2R14	199	Tatridin A	41653-75-2	TAS2R14
193	2-Heptyl-3-hydroxy-quinolone	108985-27-9	TAS2R14				
